# Synergistic Effect of Pt and Dual Ni/Co Cations in Hydrotalcite-Derived Pt/Ni_1.5_Co_0.5_AlO Catalysts for Promoting Soot Combustion

**DOI:** 10.3390/nano13040623

**Published:** 2023-02-04

**Authors:** Yilin Zhang, Peng Zhang, Jing Xiong, Yuanfeng Li, Yaxiao Ma, Sicheng Zhang, Zhen Zhao, Jian Liu, Yuechang Wei

**Affiliations:** 1 State Key Laboratory of Heavy Oil Processing, College of Science, China University of Petroleum, Beijing 102249, China; 2 Key Laboratory of Optical Detection Technology for Oil and Gas, China University of Petroleum, Beijing 102249, China

**Keywords:** hydrotalcite-derived oxides, synergistic effect, Pt, strong metal-support interaction, soot combustion

## Abstract

In this article, the catalysts of hydrotalcite-derived Ni_1.5_Co_0.5_AlO nanosheet-supported highly dispersed Pt nanoparticles (Pt_n_/Ni_1.5_Co_0.5_AlO, where n% is the weigh percentage of the Pt element in the catalysts) were elaborately fabricated by the gas-bubble-assisted membrane--reduction method. The specific porous structure formed by the stack of hydrotalcite-derived Ni_1.5_Co_0.5_AlO nanosheets can increase the transfer mass efficiency of the reactants (O_2_, NO, and soot) and the strong Pt–Ni_1.5_Co_0.5_AlO interaction can weaken the Ni/Co-O bond for promoting the mobility of lattice oxygen and the formation of surface-oxygen vacancies. The Pt_n_/Ni_1.5_Co_0.5_AlO catalysts exhibited excellent catalytic activity and stability during diesel soot combustion under the loose contact mode between soot particles and catalysts. Among all the catalysts, the Pt_2_/Ni_1.5_Co_0.5_AlO catalyst showed the highest catalytic activities for soot combustion (*T_50_* = 350 °C, TOF = 6.63 × 10^−3^ s^−1^). Based on the characterization results, the catalytic mechanism for soot combustion is proposed: the synergistic effect of Pt and dual Ni/Co cations in the Pt/Ni_1.5_Co_0.5_AlO catalysts can promote the vital step of catalyzing NO oxidation to NO_2_ in the NO-assisted soot oxidation mechanism. This insight into the synergistic effect of Pt and dual Ni/Co cations for soot combustion provides new strategies for reducing the amounts of noble metals in high-efficient catalysts.

## 1. Introduction

Diesel engines are widely used in heavy vehicles because of their low operating costs, good durability, and reliable operation under lean conditions [1]. Although diesel engines and gasoline direct-injection engines (GDI) improve fuel efficiency, the emissions of exhaust gases including NO_x_ and particulate matter (PM), lead to severe environmental pollution and human hazards [2,3]. PM can cause serious respiratory diseases and is considered to be the main source of photochemical smog and the greenhouse effect [4,5]. With increasingly stringent legislative restrictions, it is necessary to develop practical processes to remove these harmful substances [6]. The technology of diesel/gasoline particulate filters (D/GPF) combined with catalysts is one of the most effective after-treatment technologies, and D/GPF has received extensive research interest [7,8,9]. 

The main component of PM is a carbonaceous substance known as soot [10]. The soot oxidation reaction usually occurs at the three-phase contact points of soot–catalyst–gaseous reactants, which is a typical solid–solid–gas heterogeneous catalysis reaction. Thus, the soot–catalyst contact interface plays an important role in the process of catalyzing soot combustion [11,12]. It is well known that the morphology of catalysts can affect the contact efficiency between catalysts and soot particles. However, the traditional catalysts with nanopores (<25 nm) have difficulty in approaching the inner surface of the nanopores, which limits the formation of sufficient contact interfaces between soot and catalysts, thus reducing the catalytic activity for soot combustion [13]. In previously reported works, the ordered macroporous catalysts with regular pore size (~240 nm) were able to improve the contact efficiency between the catalysts and soot particles, and the three-dimensional ordered macroporous (3DOM) catalysts exhibited good catalytic activity for soot combustion [14,15,16]. Thus, it is crucial to fabricate structured macroporous catalysts for soot combustion. 

The catalyzing soot-combustion process is a typical deep-oxidation reaction, so the redox property of the catalysts can strongly affect the intrinsic catalytic activity of soot combustion. In view of the essence of catalytic soot combustion, it is a challenge to enhance the catalytic performance for soot combustion by improving the adsorption and activation abilities of catalysts for O_2_ and NO reactants. Many high-efficient catalysts for soot combustion have been reported, such as noble metals, rare earth metal oxides, and transition metal oxides [11,17,18,19]. Among these catalysts, supported noble metal catalysts have attracted extensive attention because of their abilities in improving the intrinsic redox properties of the catalysts. Noble metal (Pd, Au, and Pt) nanoparticles exhibit excellent abilities to adsorb and activate O_2_ [20,21,22,23]. The relationship between the strong noble metal and oxidesupport interaction and the catalytic activity of soot combustion is investigated by loading the noble metal onto the surface of oxides. Until now, Pt nano-catalysts have been the main active components of diesel engine exhaust purification catalysts. For Pt-supported catalysts, the utilization efficiency of Pt nanoparticles can be improved by regulating the metal-oxide/support interface structure, which is the active site derived from the strong Pt–support interaction for O_2_ adsorption and activation [24,25].

Soot combustion is a heterogeneous catalysis reaction, and the catalytic performance of noble metal catalysts is affected by many factors, including the dispersion of noble metals and the interface structure between metal nanoparticles and oxide supports [26]. The strong interaction between oxide supports and noble metal nanoparticles can induce significant changes in metal-oxide/support interface structures; thus, it can affect the catalytic oxidation ability during the oxidation reaction [26,27]. It is crucial to that the appropriate oxide supports are selected to reduce the external factors affecting the noble metal-supported catalysts and improve the catalytic activity. Layered double hydroxides (LDHs), also known as hydrotalcite-like materials, are types of anionic clay materials with a two-dimensional layered nanostructure composed of brucite-like layers and intercalated anions [28]. Due to the large specific surface area (100–300 m^2^ g^−1^), the uniform and thermally stable dispersion of metal ions, and the synergistic effect between M^2+^ and M^3+^ metal elements, the mixed metal oxides obtained by controlled thermal decomposition of LDHs have been widely used in the field of catalysis [28]. Hydrotalcite-derived oxides can act as support for dispersed metals with redox properties [29]. For example, the strong interaction between Ag nanoparticles and hydrotalcite-derived CoAl mixed oxides can increase the amounts of oxygen species at the Ag sites, promoting the intrinsic activity for soot combustion [30]. According to our previous work, the uniform dispersion of metal ions in the nanosheet of hydrotalcite-derived oxides and the specific porous structure formed by the stacking of nanosheets can promote soot combustion [31]. Thus, Pt nanoparticles supported on the surface of hydrotalcite-derived oxide supports can improve the utilization efficiency of Pt components, and further provide a way to investigate the influence of the synergistic effect between the metal ions in supports and Pt nanoparticles on the catalytic activity for soot combustion.

Herein, the catalysts of hydrotalcite-derived Ni_1.5_Co_0.5_AlO nanosheets-supported highly dispersed Pt nanoparticles (Pt_n_/Ni_1.5_Co_0.5_AlO) were elaborately fabricated by the gas-bubble-assisted membrane-reduction method. The specific porous structure formed by the stack of hydrotalcite-derived Ni_1.5_Co_0.5_AlO nanosheets can increase the transfer mass efficiency of the reactants (O_2_, NO, and soot), and the strong Pt-Ni_1.5_Co_0.5_AlO interaction can weaken the Ni/Co-O bond for promoting the mobility of lattice oxygen and the formation of surface-oxygen vacancies. Pt_n_/Ni_1.5_Co_0.5_AlO catalysts exhibited excellent catalytic activity and stability during diesel soot combustion under the loose contact mode between soot particles and catalysts. A catalytic mechanism for soot combustion is proposed: the synergistic effect of Pt and dual Ni/Co cations in Pt/Ni_1.5_Co_0.5_AlO catalysts can promote the vital step of catalyzing NO oxidation to NO_2_ in the NO-assisted soot oxidation mechanism. This process provides new strategies for reducing the amounts of noble metals in high-efficient catalysts.

## 2. Experimental Sections

### 2.1. Catalyst Preparation

**Figure 1:**
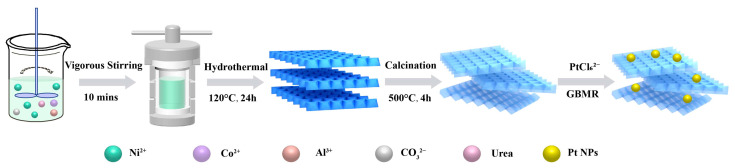
Schematic diagram of preparation for Pt_n_/Ni_1.5_Co_0.5_AlO catalysts.

The Ni_1.5_Co_0.5_Al-LDH nanosheet as the catalyst precursor was prepared by a one-step hydrothermal method. The synthesis processes are shown in Figure 1, and the details are described as follows: Ni(NO_3_)_2_·6H_2_O (0.0075 mol), Co(NO_3_)_2_·6H_2_O (0.0025 mol), Al(NO_3_)_3_·9H_2_O (0.005 mol), and urea (0.05 mol) were dissolved in 60 mL deionized water under vigorous stirring for 10 min. All of the reagents were purchased from Shanghai Macklin Biochemical Co., Ltd. (Shanghai, China) Then, the above-mentioned solution was transferred into an autoclave (100 mL) and heated at 120 °C for 24 h. The resulting precipitation was washed three times with deionized water and collected by centrifugation. After being vacuum-dried for 24 h, the Ni_1.5_Co_0.5_Al-LDH precursor was obtained. Finally, the prepared precursor was calcined at the temperature of 500 °C for 4 h; then, the product was named as the Ni_1.5_Co_0.5_AlO catalyst. The synthesis of Pt_n_/Ni_1.5_Co_0.5_AlO catalysts was carried out by gas-bubbling-assisted membrane reduction (GBMR) [14]. The schematic representation of the gas-bubbling-assisted membrane reduction (GBMR) device is exhibited in Figure S1. The GBMR equipment was assembled by our laboratory. (Beijing, China) The procedures were as follows: Ni_1.5_Co_0.5_AlO catalyst (0.5 g) was dispersed into deionized water (200 mL) under magnetic stirring at room temperature, and the stoichiometric amount of HPtCl_4_ solution was added dropwise to the above solution. The polyvinylpyrrolidone (PVP) solution ([PVP_unit_]/[Pt] = 100) as a stabilizer was added into the mixed solution. The cyclic flow of the mixed solution in a glass reactor and beaker was driven by a peristaltic pump at the rate of 300 mL min^−1^. The HPtCl_4_ and PVP reagents were purchased from Shanghai Aladdin Biochemical Technology Co., Ltd. (Shanghai, China). The NaBH_4_ reagent was purchased from Shanghai Macklin Biochemical Co., Ltd. (Shanghai, China). In the reactor, the solution mixture flowed inside the glass tube and outside the ceramic tube. With NaBH_4_ solution as the reducing agent ([NaBH_4_]/[Pt] molar ratio of 5), the reactor was injected with an 0.8 mL min^−1^ flow rate through constant current pump. When the NaBH_4_ solution passed through two ceramic tubes (3 mm × 160 mm) and penetrated into the glass tube through the pores on the tube wall (d = 40 nm), a reduction of Pt ions occurred. Through a hydrogen-bubbling-assisted stirring operation, the reaction system was subjected to further bubbling to generate highly uniform reducing agent dispersion and provide a reduction atmosphere, which was essential for the size and distribution of Pt nanoparticles. The mixture was stirred with hydrogen (40 mL min^−1^) through two other ceramic tubes until NaBH_4_ was completely consumed. After filtration, the product was washed with deionized water until the Cl^-^ was not detected by AgNO_3_. The obtained solid was further dried overnight at 50 °C and then calcined at 500 °C for 2 h in air to remove H_2_O and stabilize reagents. The calcined products were named Pt_n_/Ni_1.5_Co_0.5_AlO catalysts, where n% was the initial mass percent of the Pt element in the catalyst and the n values were 1, 2, 4, and 6. The synthesis process diagram of Pt_n_/Ni_1.5_Co_0.5_AlO catalysts is shown in Figure 1. 

### 2.2. Characterizations

The powder X-ray diffraction (XRD) measurements were detected by a Bruker D8 Advance (Bruker Axs Gmbh, Germany) X-ray diffractometer equipped with Cu/Kα radiation (λ = 1.5406 Å). The 2*θ* angle of the diffractometer was stepped from 5° to 90° at the scan rate of 5° min^−1^. The Fourier transform infrared (FT-IR) spectra were recorded by an IR Tracer-100 spectrometer (Shimadzu, Japan) using the KBr disk technique. The morphology and microstructure of as-prepared Pt_n_/Ni_1.5_Co_0.5_AlO catalysts were investigated by a scanning electron microscope (SEM, FEI Quanta 200F, FEI Company, Eindhoven, Holland) and a transmission electron microscope (TEM, JEOL JEM 2100, JEOL company, Japan). The surface properties of Pt_n_/Ni_1.5_Co_0.5_AlO catalysts were investigated by using a Perkin–Elmer PHI-1600 ESCA X-ray photoelectron spectroscope (XPS, Massachusetts, USA) equipped with a monochromatic Mg-Kα X-ray source. The Brunouer–Emmett–Teller (BET) method was selected to obtain the specific surface area of Pt_n_/Ni_1.5_Co_0.5_AlO catalysts. Raman spectra were measured by an InVia Reflex-Ranisho spectrometer(Renishaw, United Kingdom) in the anti-Stokes range of 200 to 1300 cm^−1^ and the samples were excited by a He-Ge laser at 532 nm. A chemical adsorption apparatus (Autosorb IQ Quantachrome, State of California, USA) was chosen to measure the experiments of H_2_-temperature-programmed reduction (H_2_-TPR) for consumption of hydrogen. NO temperature-programmed oxidation (NO-TPO) was carried out on a fixed-bed reactor. The catalyst (0.1 g) was pretreated with N_2_ at 100 °C for 30 min, then heated at 2 °C min^−1^ from 100 to 450 °C. The air flow through the catalysts at 50 mL min^−1^ consisted of O_2_ (5 vol %), NO (0.2 vol %), and N_2_ as the equilibrium atmosphere. In situ diffuse infrared Fourier transform spectra (DRIFTS) were measured on an IR Tracer-100 spectrometer (Shimadzu, Japan) equipped with liquid-nitrogen-cooled MCT detectors. The catalysts were pretreated at 200 °C for 1 h under N_2_ atmosphere, then cooled to 50 °C to collect background. The experiments were carried out under an ambient reaction containing O_2_ (5 vol %) and NO (0.2 vol %) balanced with N_2_ at a flow rate of 50 mL min^−1^. The spectra were recorded to track surface species changes at 50 °C intervals from 50 to 400 °C. 

### 2.3. Catalytic Performance Evaluation

The catalytic activities of Pt_n_/Ni_1.5_Co_0.5_AlO catalysts for soot combustion were evaluated for temperature-programmed oxidation (TPO) in a fixed-bed tubular quartz system and each soot-TPO was operated at a heating rate of 2 °C min^−1^ from 150 to 650 °C. The soot particles were commercial carbon particles (Printex-U, average diameter of 25 nm) purchased from Degussa and used to simulate soot particles in diesel exhaust [32]. The catalyst (100 mg) and soot (10 mg) were uniformly mixed in an agate bowl at the weight ratio of 10:1 to form the loose contact mode. The gas reactant containing O_2_ (5%), NO (0.2%), and Ar as equilibrium gas proceeded through the mixture of catalyst and soot at a flow rate of 50 mL min^−1^. The gas products at the export were analyzed by online GC with an FID detector (GC 9890, Shanghai Sida Analytical Instrument Co., Ltd., Shanghai). The catalytic soot combustion activity was estimated by the values of *T_10_, T_50_*, and *T_90_*, which were defined as the temperatures at 10%, 50%, and 90% of soot conversion, respectively. The selectivity of generated gas to CO_2_ (S_CO2_) was defined by the equation: S_CO2_ = C_CO2_/(C_CO_ + C_CO2_), and S_CO2_^m^ was defined as S_CO2_ at the maximum of C_CO2_. The turnover frequency (TOF) of the catalysts as the intrinsic activity was defined as the ratio of the isothermal reaction rate (R) to the active oxygen density (D_o_) of the active sites. The isothermal reaction rates of soot oxidation were obtained under a stable low conversion of soot particles. Since the conversion rates of soot were low (<10%) and the reaction rates were almost constant, the isothermal oxidation of soot was carried out at 300 °C. The surface density of active sites was acquired by the isothermal anaerobic titration at 300 °C. 

## 3. Results

### 3.1. XRD Analyses

**Figure 2:**
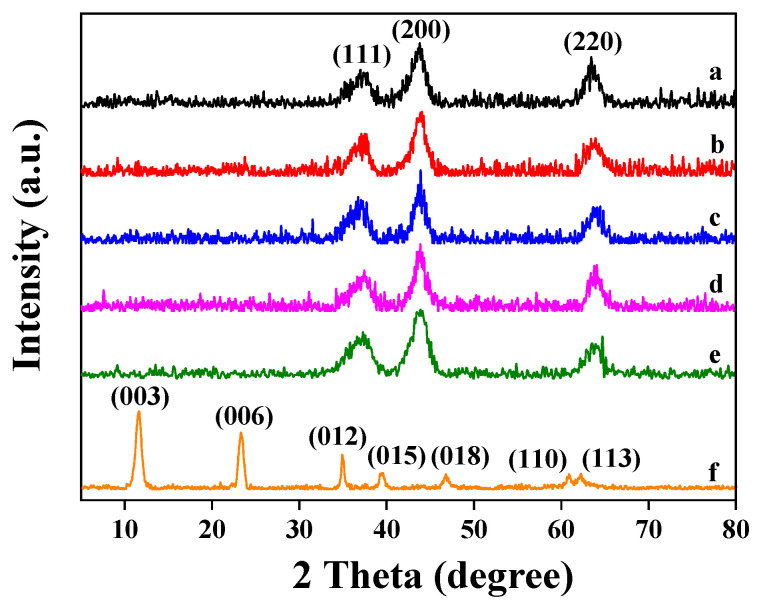
XRD patterns of Ni_1.5_Co_0.5_Al-LDH precursor and the Pt_n_/Ni_1.5_Co_0.5_AlO catalysts: (a) Ni_1.5_Co_0.5_AlO; (b) Pt_1_/Ni_1.5_Co_0.5_AlO; (c) Pt_2_/Ni_1.5_Co_0.5_AlO; (d) Pt_4_/Ni_1.5_Co_0.5_AlO; (e) Pt_6_/Ni_1.5_Co_0.5_AlO; (f) Ni_1.5_Co_0.5_Al-LDH.

To investigate the crystal phase structure of the Pt_n_/Ni_1.5_Co_0.5_AlO catalysts, powder X-ray diffraction was carried out, and the results are exhibited in Figure 2. As shown in Figure 2, the typical diffraction peaks of the Ni_1.5_Co_0.5_Al-LDH precursor are located at 11.5°, 23.2°, 34.8°, 39.4°, 46.6°, 60.7°, and 62.2°, which are assigned to the (003), (006), (012), (015), (018), (110), and (113) crystal planes of hydrotalcite phase (JPCDS 51-0045), respectively [33]. There are no characteristic peaks of Co(OH)_2_ shown in Figure 2, indicating that Co ions were successfully intercalated into NiAl hydrotalcite layers. After being calcined at 500 °C, the Ni_1.5_Co_0.5_Al-LDH was transformed to Ni_1.5_Co_0.5_Al-oxide (Ni_1.5_Co_0.5_AlO), mainly consisting of Ni-related phases, characterized by diffraction peaks at 37.2°, 43.7° and 63.5° corresponding to the (111), (200), and (220) crystal planes of cubic NiO (JCPDS 47-1049), respectively [34]. However, no characterized peaks of the Al_2_O_3_ phase are shown in Figure 2, suggesting that the Al ions replaced the positions of the Ni ions and dispersed in the hydrotalcite nanosheets. The Co-based and Ni-based oxides may co-exist in the Ni_1.5_Co_0.5_AlO catalyst, and Co ions can partially replace the Ni ions in the NiO lattice [31]. After loading the Pt nanoparticles, the Pt_n_/Ni_1.5_Co_0.5_AlO catalysts exhibited three peaks similar with those of Ni_1.5_Co_0.5_AlO, while no crystalline of Pt species were detected, which is ascribed to the small crystalline size and good dispersion of the Pt nanoparticles outside the detection limit of XRD spectra [35]. 

### 3.2. N_2_ Adsorption-Desorption Experiments 

**Figure 3:**
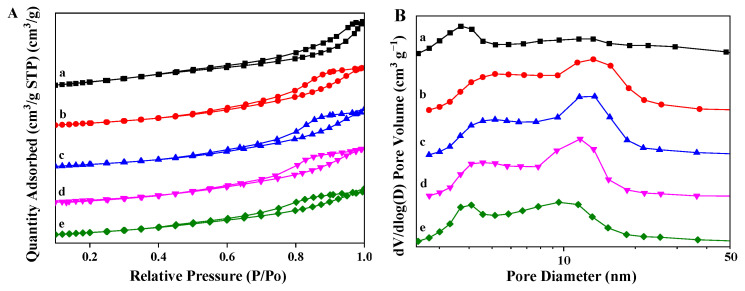
Nitrogen adsorption–desorption isotherms (**A**) and pore-size distribution curves (**B**) of Pt_n_/Ni_1.5_Co_0.5_AlO catalysts: (a) Ni_1.5_Co_0.5_AlO; (b) Pt_1_/Ni_1.5_Co_0.5_AlO; (c) Pt_2_/Ni_1.5_Co_0.5_AlO; (d) Pt_4_/Ni_1.5_Co_0.5_AlO; (e) Pt_6_/Ni_1.5_Co_0.5_AlO.

As shown in Figure 3A, the N_2_ adsorption–desorption isotherms of the Pt_n_/Ni_1.5_Co_0.5_AlO catalysts exhibit the characteristic type-IV adsorption–desorption isotherms with an H3-hysteresis loop (P/P_0_ = 0.8–1.0). This type of adsorption isotherm represents mesoporous materials with no or few micropores, and there is the strong interaction between adsorbent and adsorbate molecules [36]. In the pore-size distribution curves displayed in Figure 3B, the majority of pores fall in the meso-range scale and the average pore diameter ranges from 6 to 8 nm in Table S1. The specific surface area of Pt_n_/Ni_1.5_Co_0.5_AlO catalysts were measured by BET methods in Table S1. As shown in Table S1, the surface area of pure Ni_1.5_Co_0.5_AlO support is 189.06 m² g^−1^, while the surface area of Pt-supported catalysts decreased in the range of 122–125 m² g^−1^. The total pore volume exhibited a similar tendency. These variations are attributed to the fact that the partial filling of Pt nanoparticles leads to the narrowing of mesopores accompanied by a decrease in specific surface area [37]. It is noted that the Pt-based catalysts rarely changed, suggesting that the Pt nanoparticles are well dispersed on the surface of the Ni_1.5_Co_0.5_AlO support.

### 3.3. FTIR and Raman Spectra 

**Figure 4:**
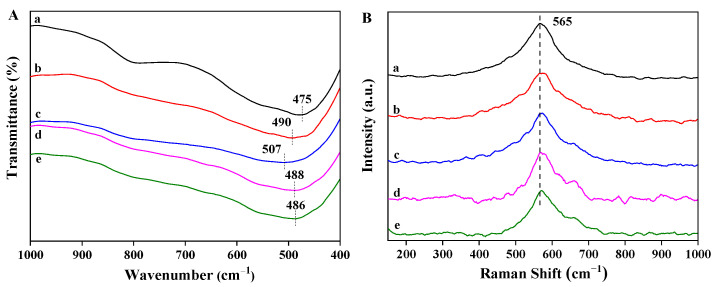
FT-IR spectra (**A**) and Raman spectra (**B**) of Pt_n_/Ni_1.5_Co_0.5_AlO catalysts: (a) Ni_1.5_Co_0.5_AlO; (b) Pt_1_/Ni_1.5_Co_0.5_AlO; (c) Pt_2_/Ni_1.5_Co_0.5_AlO; (d) Pt_4_/Ni_1.5_Co_0.5_AlO; (e) Pt_6_/Ni_1.5_Co_0.5_AlO.

To explore the effect of Pt nanoparticles on the phase structure of Ni_1.5_Co_0.5_AlO support, FT-IR and Raman spectra were carried out, and the results are shown in Figure 4. As shown in Figure 4A, the obvious vibration peak located at 475 cm^−1^ is assigned to the vibration of Ni-O bond [38]. However, the FTIR spectrum of Ni_1.5_Co_0.5_AlO catalyst is similar to that of the Ni-based oxides, as described in the previous work, which has slightly shifted in comparison with those of pure NiO (450 cm^−1^) [39]. There are no additional vibration peaks of Co-based oxides and Al_2_O_3_ observed in the spectra, indicating that the Co and Al ions are embedded into the Ni_1.5_Co_0.5_AlO lattice, resulting in the shift of the vibration peak [31]. After the introduction of supported Pt nanoparticles, the vibration peak shifts toward to high wavenumber, which is related to the distribution of the electron state at the interface between the supported Pt nanoparticles and the oxide support [40]. 

The molecular structure of the Pt_n_/Ni_1.5_Co_0.5_AlO catalysts was further investigated by Raman spectra using a laser with an excitation wavelength of 532 nm. As exhibited in Figure 4B, for Ni_1.5_Co_0.5_AlO support, one Raman band is observed at 565 cm^−1^, which is assigned to the stretching vibration of Ni-O bonds [41]. Since parts of the Co ions and the Al ions replace the positions of the Ni ions in the NiO crystal lattice, the Raman peak of the Ni_1.5_Co_0.5_AlO catalyst deviates from that of the pure NiO phase [31]. We observed that the Raman peak at 565 cm^−1^ for Ni_1.5_Co_0.5_AlO catalyst slightly shifted to high frequency after the loading of the Pt nanoparticles (in Figure S2), which is ascribed to the strong Pt-Ni_1.5_Co_0.5_AlO interaction resulting in the surface lattice distortion of Ni_1.5_Co_0.5_AlO at the interface between Pt and Ni_1.5_Co_0.5_AlO [25]. This result was consistent with FTIR. The strong interaction between Pt nanoparticles and support may affect the catalytic activity, which will be discussed in the following sections. Combined with the results of XRD, FTIR, and Raman, it demonstrates that Co ions can replace the positions of Ni ions in the Ni_1.5_Co_0.5_AlO catalyst, rather than form a separate phase, which is closely related to the redox activity of the Ni_1.5_Co_0.5_AlO catalyst.

### 3.4. SEM and TEM Images

**Figure 5:**
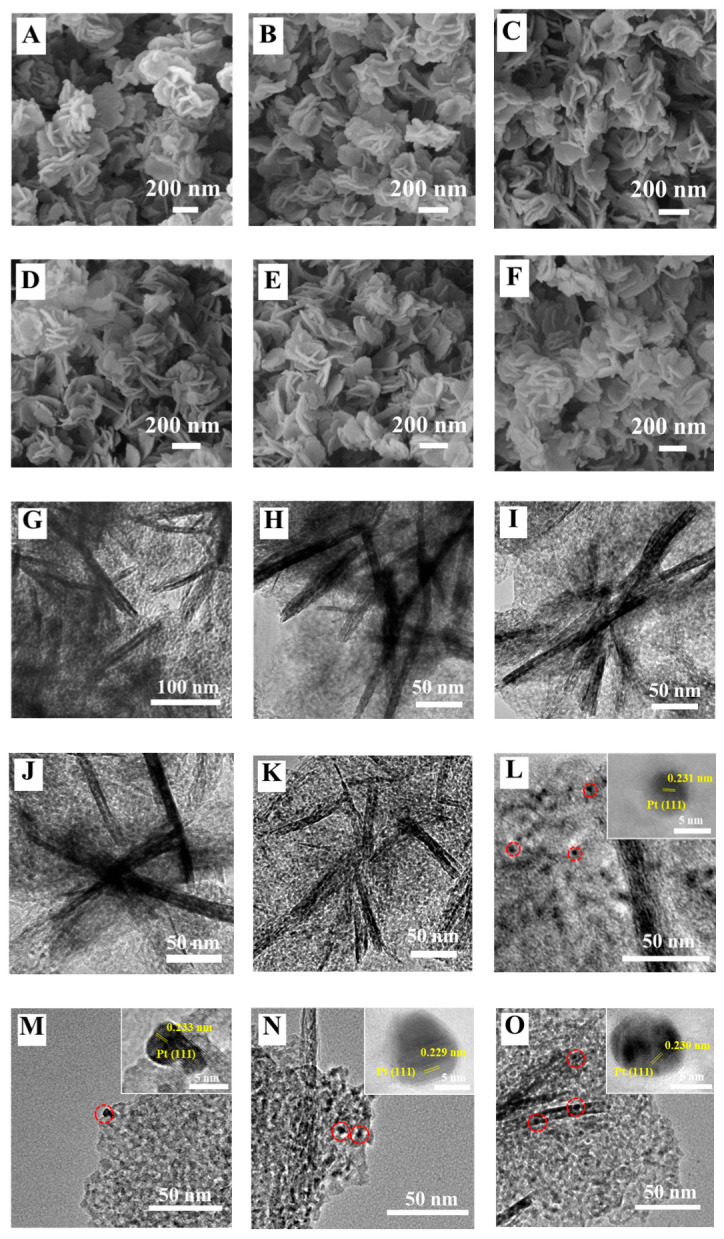
SEM (**A**–**F**) and TEM (**G**–**O**) images of Pt_n_/Ni_1.5_Co_0.5_AlO catalysts: (**A**) Ni_1.5_Co_0.5_Al-LDH; (**B**,**G**) Ni_1.5_Co_0.5_AlO; (**C**,**H**,**L**) Pt_1_/Ni_1.5_Co_0.5_AlO; (**D**,**I**,**M**) Pt_2_/Ni_1.5_Co_0.5_AlO; (**E**,**J**,**N**) Pt_4_/Ni_1.5_Co_0.5_AlO; (**F**,**K**,**O**) Pt_6_/Ni_1.5_Co_0.5_AlO.

To investigate the microstructure and morphology of the Pt_n_/Ni_1.5_Co_0.5_AlO catalysts, the scanning electron microscope and transmission electron microscope were used and the images are shown in Figure 5. As depicted in Figure 5A, the regular nanosheets stacking structure of the Ni_1.5_Co_0.5_Al-LDH precursors were observed. After being calcined at 500 °C, the Ni_1.5_Co_0.5_AlO catalyst still maintained the regular nanosheets stacking structure, which is observed in Figure 5B. With the increase in the loading amount of Pt nanoparticles, the regular nanosheets stacking structure of the Pt_n_/Ni_1.5_Co_0.5_AlO catalysts remained unchanged, as shown in Figure 5C–F, indicating that the Pt nanoparticles were uniformly dispersed at the support without destroying the morphology of the support. The results are consistent with those of XRD and BET. According to our previous report, these open macropores constructed by nanosheet stacking can increase the transfer mass efficiency among the reactants (O_2_, NO, and soot) under the gas reactant flow [31]. Thus, the Ni_1.5_Co_0.5_AlO support with open macropores and Pt nanoparticles is beneficial to boost soot combustion. 

The microstructure of Pt_n_/Ni_1.5_Co_0.5_AlO catalysts was recognized in TEM images, and the clear lattice fringes were seen in the inset of the HRTEM images. As shown in Figure 5G–K, all of the Pt_n_/Ni_1.5_Co_0.5_AlO catalysts exhibited regular nanosheet morphology, further suggesting that the loading processes of the Pt nanoparticles and calcination treatment rarely affected the nanosheet structure. The average size of nanosheet distribution was obviously uniform, in the range of 100–200 nm. As shown in Figure 5L–O, the Pt nanoparticles were evenly distributed on the surface of the Pt_n_/Ni_1.5_Co_0.5_AlO catalysts and exhibited similar spherical morphologies. The lattice fringe of those catalysts, shown inset of Figure 5L–O, was ~2.3 Å, which belongs to the FCC (111) crystal face of Pt [25]. The particle-size distribution of the Pt nanoparticles was in the range of 5–8 nm. From the results of the SEM and TEM, we noted that the Pt nanoparticles were homogeneously dispersed on the surface of the Ni_1.5_Co_0.5_AlO nanosheets, which facilitated the investigation of the synergistic effect between Pt and the support on the catalytic performance for soot combustion. 

### 3.5. H_2_-TPR Profiles 

**Figure 6:**
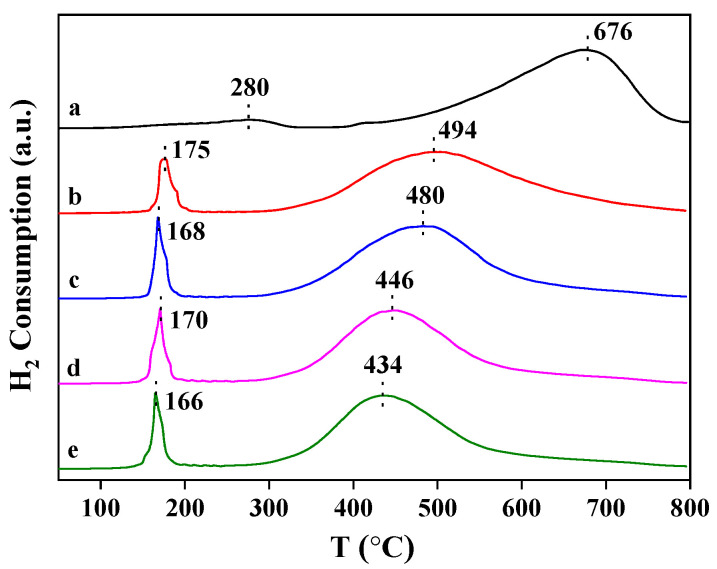
H_2_-TPR profiles of Pt_n_/Ni_1.5_Co_0.5_AlO catalysts: (a) Ni_1.5_Co0.5AlO; (b) Pt_1_/Ni_1.5_Co_0.5_AlO; (c) Pt_2_/Ni_1.5_Co_0.5_AlO; (d) Pt_4_/Ni_1.5_Co_0.5_AlO; (e) Pt_6_/Ni_1.5_Co_0.5_AlO.

In a deep oxidation reaction, the redox property of the catalysts is a crucial factor in evaluating catalytic performance, which is usually measured by a temperature-programmed reduction of H_2_. The variation in the redox properties of the Pt_n_/Ni_1.5_Co_0.5_AlO catalysts, after the introduction of supported Pt nanoparticles, is shown in Figure 6. The Ni_1.5_Co_0.5_AlO catalyst exhibited two reduction peaks. The characterization results verified that the Co ions can replace the positions of the Ni ions in the Ni_1.5_Co_0.5_AlO catalyst. The low reduction peak located at 280 °C was assigned to the reduction of Co^3+^ to Co^2+^, while the high reduction peak that emerged at 676 °C was attributed to the reduction of Co^2+^ to Co^0^ and Ni^2+^ to Ni^0^ [31]. After the loading of the Pt nanoparticles, there were two ranges at a low temperature region (<200 °C) and a high temperature region (>400 °C). The reduction peak that emerged at ~170 °C was attributed to the reduction of the Pt oxides or surface active oxygen species at the Pt-Ni_1.5_Co_0.5_AlO interfaces [42]. The Pt_n_/Ni_1.5_Co_0.5_AlO catalysts with different loading capacities of Pt nanoparticles exhibited similar reduction peaks at the low temperature region (<200 °C), suggesting the same interaction between Pt and oxides. As the loading amounts of Pt increased gradually, the reduction peak located at the high temperature region (>400 °C) shifted toward low temperature. This result suggests that Pt nanoparticles can enhance the redox ability of Ni_1.5_Co_0.5_AlO support, which may be attributed to the strong interaction between Pt and the Ni_1.5_Co_0.5_AlO catalyst, resulting in the increased mobility of lattice oxygen and the oxygen vacancies at the metal-oxide/support interface. Consequently, this will be beneficial in improving the catalytic performance for soot combustion. 

### 3.6. The Results of XPS Spectra 

**Figure 7:**
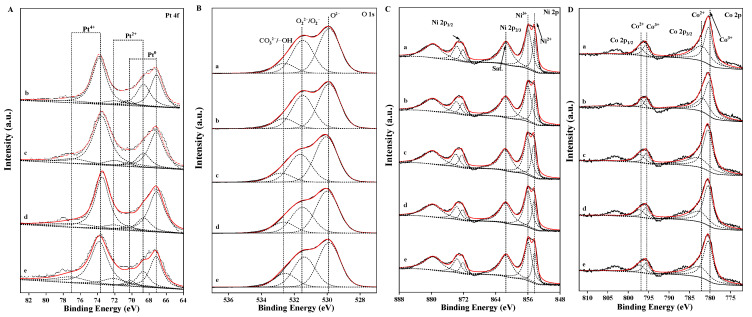
XPS spectra of Pt 4f (**A**), O 1s (**B**), Ni 2p (**C**), and Co 2p (**D**) regions over Pt_n_/Ni_1.5_Co_0.5_AlO catalysts: (a) Ni_1.5_Co_0.5_AlO; (b) Pt_1_/Ni_1.5_Co_0.5_AlO; (c) Pt_2_/Ni_1.5_Co_0.5_AlO; (d) Pt_4_/Ni_1.5_Co_0.5_AlO; (e) Pt_6_/Ni_1.5_Co_0.5_AlO.

The XPS spectra of the Pt_n_/Ni_1.5_Co_0.5_AlO catalysts were measured to investigate the surface element component, the metal oxides states, and the adsorbed oxygen species. In Figure 7A, the Pt 4f spectra of all catalysts are exhibited to explore the strong interaction between Pt and Ni_1.5_Co_0.5_AlO. The Pt 4f region was decomposed into three components by standard deconvolution: the binding energy peaks at ~67.2 and ~70.5 eV were assigned to the metallic Pt species, while the corresponding peaks located at ~68.7, ~72.1, ~73.5, and 76.8 eV were assigned to the Pt^2+^ and Pt^4+^ species, respectively. The binding energy of Pt located at 67.2 eV was ascribed to the the appearance of new surface interactions on the catalyst and was somewhat related to the synthetic method [43,44]. This indicated that both the metallic and ionic Pt species were present on the surface of the Pt_n_/Ni_1.5_Co_0.5_AlO catalysts. The formation of cationic-state Pt species resulted from the electron transfer from Pt^0^ to Ni_1.5_Co_0.5_AlO at the Pt-support interface, which was attributed to the strong interaction between Pt and Ni_1.5_Co_0.5_AlO support. Ionic Pt species are crucial factors for soot combustion, which is attributed to its dramatic adsorption and activation ability for O_2_ and NO [45]. The chemical state and quantitative analysis results are listed in Table S2. The molar ratios of surface (Pt^2+^ + Pt^4+^)/Pt^0^ species were calculated by the areas of the corresponding split peaks in the XPS spectra. As shown in Table S2, the Pt species ratio (*R^a^*) over the Pt_1_/Ni_1.5_Co_0.5_AlO catalyst was about 2.145, which was the lowest *R^a^* value among all the catalysts. With the increase in the supported Pt content, the *R^a^* value increased, indicating that the activation ability for oxygen for the Pt-supported catalysts may be related to the content of the Pt species. 

The density of surface-active oxygen species is closely related to the catalytic performance during the deep oxidation reaction. To observe the distribution of surface oxygen species over the Pt_n_/Ni_1.5_Co_0.5_AlO catalysts, the XPS spectra of O 1s were measured by the peak-fitting method, and the results are shown in Figure 7B. As depicted in Figure 7B, the asymmetric O 1s spectra of the catalysts were deconvoluted into three types of oxygen species: lattice oxygen species (O^2−^), active oxygen species (O_2_^2−^/O_2_^−^, and CO_3_^2−^/-OH), with binding energies of 529.9, 531.5, and 532.6 eV, respectively [46,47,48]. Based on the relative content of the oxygen species, the molar ratios of the adsorbed oxygen species to the lattice oxygen species were calculated. The molar ratio values (*R^d^*) of the oxygen species/lattice oxygen species over the Pt_n_/Ni_1.5_Co_0.5_AlO catalysts are listed in Table S2. As shown in Table S2, the *R^d^* value of pure Ni_1.5_Co_0.5_AlO catalyst was only 1.004. After the introduction of supported Pt nanoparticles, the *R^d^* values of the Pt_n_/Ni_1.5_Co_0.5_AlO catalysts were obviously higher than that of the Ni_1.5_Co_0.5_AlO support. With the increase in Pt content, the *R* values of the Pt_n_/Ni_1.5_Co_0.5_AlO catalysts increased. Among all the catalysts, the Pt_2_/Ni_1.5_Co_0.5_AlO catalyst possessed the highest value, suggesting that the amounts of surface-active oxygen species were higher than other catalysts. This result is in accordance with that of the Pt species. The strong interaction between Pt nanoparticles and Ni_1.5_Co_0.5_AlO support over the Pt_n_/Ni_1.5_Co_0.5_AlO catalysts enhanced the adsorption and activation property for oxygen, which can promote the catalytic performance for soot combustion.

In order to further study the strong interaction between Pt nanoparticles and Ni_1.5_Co_0.5_AlO support, the XPS spectra of Ni, Co, and Al elements over the Pt_n_/Ni_1.5_Co_0.5_AlO catalysts were also measured. The XPS Ni 2p spectra of the Pt_n_/Ni_1.5_Co_0.5_AlO catalysts are depicted in Figure 7C; there are two strong peaks centered at 854.9 eV and 872.8 eV, which corresponds to the spin-orbit splitting into Ni 2p_3/2_ and Ni 2p_1/2_, respectively [31]. The peaks located at 861.4 eV and 879.5 eV were assigned to the satellite peaks of Ni 2p_3/2_ and Ni 2p_1/2_, respectively. Notably, the spectra of Ni 2p_3/2_ were asymmetric and deconvoluted into two characteristic peaks, which were assigned to Ni^3+^ (855.9 eV) and Ni^2+^ (854.3 eV), respectively [49]. The molar ratios of surface Ni^3+^/Ni^2+^ were calculated by the areas of characteristic deconvolution peaks, and the relative values are exhibited in Table S2. However, the values (*R^b^*) of Ni^3+^/Ni^2+^ decreased with increases in Pt content. Among all the Pt_n_/Ni_1.5_Co_0.5_AlO catalysts, the Pt_2_/Ni_1.5_Co_0.5_AlO catalyst had the smallest *R^b^* value (1.801), while the *R^b^* value of the pure Ni_1.5_Co_0.5_AlO catalyst was 2.021. This is attributed to the fact that the supported Pt species mainly exist in high-valence states, and part of the Ni^3+^ in the Ni_1.5_Co_0.5_AlO catalyst is converted to Ni^2+^ to balance the valence states. The Ni^3+^/Ni^2+^ cycle ion pairs can promote the electron transfer at the interface of Pt and the Ni_1.5_Co_0.5_AlO catalyst [50]. It is noteworthy that, with further increases in Pt content, the *R^b^* values of Ni^3+^/Ni^2+^ slightly increased. This tendency corresponded to those of Pt species, suggesting that a close relationship exists between Pt and Ni components. The strong Pt–support interaction can weaken the metal-O and enhance the activation of lattice oxygen at the Pt–Ni_1.5_Co_0.5_AlO interface, which is beneficial to the formation of oxygen vacancies at the interface of Pt–support. 

The Co 2p XPS spectra are shown in Figure 7D, which exhibits two major peaks located at ~780.5 eV and ~795.6 eV, which correspond to the Co 2p_3/2_ and Co 2p_1/2_ spin-orbital peaks, respectively [51]. The signals of the Co^2+^ and Co^3+^ species were observed after the standard deconvolution of Co 2p spectra. The peaks centered at ~779.8 eV and ~795.4 eV were assigned to the characterized peaks of the Co^3+^ species, while the peaks centered at ~781.9 eV and ~796.8 eV were assigned to the Co^2+^ species, which is attributed to the spin-orbit splitting of the Co^3+^ and Co^2+^ species into Co 2p_3/2_ and Co 2p_1/2_ in the Pt_n_/Ni_1.5_Co_0.5_AlO catalysts, respectively [52]. On the basis of corresponding split peak area in the XPS spectrum, the molar ratio (*R^b^*) of the Co^3+^/Co^2+^ species over the catalysts was calculated and the values are shown in Table S2. The pure Ni_1.5_Co_0.5_AlO possesses the highest value (1.732) of the Co^3+^/Co^2+^ species. After the introduction of the Pt nanoparticles, the molar ratio values of Co^3+^/Co^2+^ decreases with the increase in Pt content, suggesting that Pt atoms affected the electron state distribution of Co atoms at the interface of the Pt nanoparticles and the support. Notably, among all the catalysts, the Pt_2_/Ni_1.5_Co_0.5_AlO catalyst possessed the lowest value of Co^3+^/Co^2+^ (1.653). This tendency was consistent with those of the Ni species, suggesting that a synergistic effect exists between Pt and dual Ni/Co cations. Moreover, the Al 2p spectra exhibited only a single peak that emerged at ~73.7 eV, as shown in Figure S3, which is lower than that of Al_2_O_3_ (74.1 eV). This verified that Al^3+^ ions can substitute for the Ni^2+^ ions in Ni_1.5_Co_0.5_AlO oxides, which is consistent with the results of XRD. After the introduction of Pt nanoparticles, the peak patterns rarely changed. In conclusion, Pt atoms at the metal-oxide interface mainly exist as positively charged Pt^&+^ species, and the strong interaction between Pt nanoparticles and Ni_1.5_Co_0.5_AlO supports can weaken the Ni/Co-O bonds to increase the mobility of lattice oxygen and promote the formation of oxygen vacancies to adsorb and activate O_2_ for enhancing the catalytic performances of soot oxidation. 

### 3.7. Catalytic Performances for Soot Combustion

**Figure 8:**
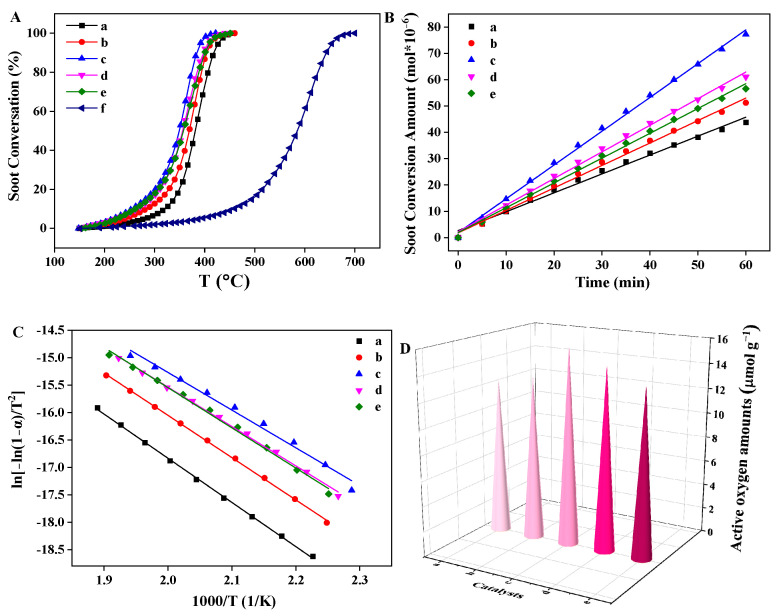
Catalytic performances for soot-conversion percentage (**A**), soot-conversion amount as a function of time (**B**), Arrhenius plots (**C**), and active oxygen amount (**D**) over Pt_n_/Ni_1.5_Co_0.5_AlO catalysts: (a) Ni_1.5_Co_0.5_AlO; (b) Pt_1_/Ni_1.5_Co_0.5_AlO; (c) Pt_2_/Ni_1.5_Co_0.5_AlO; (d) Pt_4_/Ni_1.5_Co_0.5_AlO; (e) Pt_6_/Ni_1.5_Co_0.5_AlO; (f) no catalyst.

The catalytic activities of the Pt_n_/Ni_1.5_Co_0.5_AlO catalysts for combustion were evaluated by soot-temperature-programmed oxidation (soot-TPO) under the loose contact between soot particles and catalysts. The results are exhibited in Figure 8 and Table 1. The soot-TPO profile of pure soot particles without catalysts was also measured to compare the activities of the catalysts. As shown in Figure S4, we observed that the peak temperature of the CO_2_ concentration curve for pure soot particles was higher than 600 °C. Its values of *T_50_* and *S*_CO2_^m^ were 596 °C and 65.2%, respectively. Notably, after the introduction of the Pt_n_/Ni_1.5_Co_0.5_AlO catalysts, the peak temperature of the CO_2_ concentration curve shifted toward low temperature during catalytic soot combustion, indicating that all the catalysts exhibited preferable catalytic activity for soot combustion and the selectivity of the CO_2_ product was promoted to almost 100%, as shown in Table 1. 

As shown in Figure 8A and Table 1, the Ni_1.5_Co_0.5_AlO support exhibited high catalytic activity for soot combustion, and its values of *T_10_*_,_ *T_50_*, and *T_90_* were 320, 380, and 419 °C, in comparison with the pure soot particles. According to our previous reports, the hydrotalcite-like nanosheet structure can promote mass transport efficiency between soot particles and reaction gas (NO and O_2_) [31]. As depicted in the SEM images, it can be observed that the Ni_1.5_Co_0.5_AlO support maintains a complete nanosheet structure after Pt nanoparticles are loaded, indicating that the regular morphologies can increase the contact efficiency between the catalysts and the soot particles, thereby improving the catalytic activity. In Table 1, all the Pt_n_/Ni_1.5_Co_0.5_AlO catalysts show higher catalytic activities than those with Ni_1.5_Co_0.5_AlO support. The Pt_1_/Ni_1.5_Co_0.5_AlO catalyst exhibited excellent catalytic activity in boosting soot combustion, and its values of *T_10_*, *T_50_*, and *T_90_* were 287, 368 and 404 °C, respectively. With the increase in Pt content, the values of *T_10_*, *T_50_*, and *T_90_* shifted toward low temperature. Among them, the Pt_2_/Ni_1.5_Co_0.5_AlO catalyst showed the best catalytic performance for soot combustion, with values of *T_10_*, *T_50_*, and *T_90_* are 260, 350, and 383 °C, respectively. This result is in accordance with that of XPS. The strong interaction between Pt nanoparticles and Ni_1.5_Co_0.5_AlO support weakens the Ni/Co-O bond to increase the mobility of lattice oxygen and promote the formation of oxygen vacancies. Thus, Pt and dual Ni/Co cations in the Pt/ Ni_1.5_Co_0.5_AlO catalysts achieve synergistic catalytic soot combustion. With further increases in Pt content, the position and intensity of the CO_2_ concentration peaks in the high temperature region had no obvious change, as shown in Figure S4, suggesting that there was an optimal amount of Pt nanoparticles supported on Ni_1.5_Co_0.5_AlO support. The highest selectivity of CO_2_ (S_CO2_^m^) over the Pt_n_/Ni_1.5_Co_0.5_AlO catalysts was significantly higher than that of the support, which was closely to 100%, as shown in Table 1. It indicates that the CO gas emitted from the motor engine can be oxidized immediately, which is attributed to the strong oxidation abilities of the supported Pt nanoparticles, thus avoiding atmospheric pollution. As depicted from Figure S7, the *T_50_* value of Ni_1.5_Co_0.5_AlO support shifted from 380 to 470 °C and the intensity of CO_2_ concentration curve decreased obviously with the absence of NO gas, suggesting that the catalytic oxidation of NO to NO_2_ is the essential step to improve catalytic activity during soot combustion, and further verifying the NO_2_-assisted mechanism in the process of catalytic oxidation for soot particles [7,53].

The intrinsic activities of the Pt_n_/Ni_1.5_Co_0.5_AlO catalysts for soot combustion can be evaluated by turnover frequency (TOF) values, which are obtained by the ratios of reaction rates (*R*) to the active oxygen density (D_o_) of the catalysts. The isothermal oxidation and isothermal anaerobic titration reactions were measured at 300 °C. The curve of soot conversion versus reaction time is shown in Figure S5. Figure 8B and Table 1 show that the *R* value of pure Ni_1.5_Co_0.5_AlO support was 0.12 µmol g^−1^ s^−1^, while the *R* values of the Pt-supported catalysts obviously increased, suggesting that the Pt nanoparticles can dramatically improve the catalytic performance of Ni_1.5_Co_0.5_AlO support for soot oxidation. The active oxygen amounts of the Pt-supported Ni_1.5_Co_0.5_AlO catalysts were higher than that of pure Ni_1.5_Co_0.5_AlO support. It indicates that supported Pt nanoparticles can increase reactive oxygen species at the metal-support interfaces. The synergistic effect of Pt and dual Ni/Co cations in Pt_n_/Ni_1.5_Co_0.5_AlO catalysts facilitated the adsorption–activation properties for gas reactants (NO and O_2_). Based on the ratios of the reaction rates to the active oxygen amounts of the catalysts, the values of TOF are listed in Table 1. The Pt-supported catalysts possessed larger TOF values, showing higher intrinsic activities than those of pure Ni_1.5_Co_0.5_AlO support during soot combustion. Among all the catalysts, the Pt_2_/Ni_1.5_Co_0.5_AlO catalyst possessed the largest TOF values (6.63 × 10^−3^ s^−1^), in accordance with the catalytic performance during the soot-TPO measurements. These results verified that the Pt_n_/Ni_1.5_Co_0.5_AlO catalysts exhibited excellent catalytic activities for boosting soot combustion. The catalysts for vehicle exhaust purification should possess strong stabilities in real environments. The Pt_2_/Ni_1.5_Co_0.5_AlO catalyst with the highest catalytic activity was selected for examination in three recycling soot-TPO experiments. The results are exhibited in Figure S6. After three cycles, the values of *T_10_*, *T_50_*, and *T_90_* were almost unchanged and were always within a certain range, indicating that the Pt_2_/Ni_1.5_Co_0.5_AlO catalyst possessed excellent stability during the process of soot combustion.

In addition, based on the Coats–Redfern integral method, the apparent activation energy (*E_a_*) for evaluating the catalytic performance during the soot purification process was calculated [54]. The *E_a_* values of the Pt_n_/Ni_1.5_Co_0.5_AlO catalysts are shown in Figure 8C and Table S3. The linear regression parameters (*R^2^*) of the whole Arrhenius plots were higher than 0.95, further confirming the reliability of the calculation results. The *E_a_* value of the Ni_1.5_Co_0.5_AlO catalyst was 66.6 kJ mol^−1^. After the introduction of the Pt nanoparticles, the *E_a_* value decreased to the range of 57.1–64.1 kJ mol^−1^, suggesting that the Pt-supported Ni_1.5_Co_0.5_AlO catalysts have lower energy barriers, in contrast to the pure Ni_1.5_Co_0.5_AlO support. Among all the catalysts, the Pt_2_/Ni_1.5_Co_0.5_AlO catalyst exhibited the lowest *E_a_* value (57.1 kJ mol^−1^), which was consistent with the best catalytic performance for soot combustion and confirmed the significant dependence of catalytic activity on Pt nanoparticle loading. The active oxygen amounts over the Pt_n_/Ni_1.5_Co_0.5_AlO catalysts are exhibited in Figure 8D and Table 1. It is noted that the Pt_2_/Ni_1.5_Co_0.5_AlO catalyst possesses the highest active-oxygen amounts, suggesting that the Pt_2_/Ni_1.5_Co_0.5_AlO catalyst had the best catalytic performance during soot combustion. This result is in accordance with the soot-TPO measurement, indicating that there is a close relationship between catalytic activity for soot combustion and active-oxygen amounts. 

**Figure 9:**
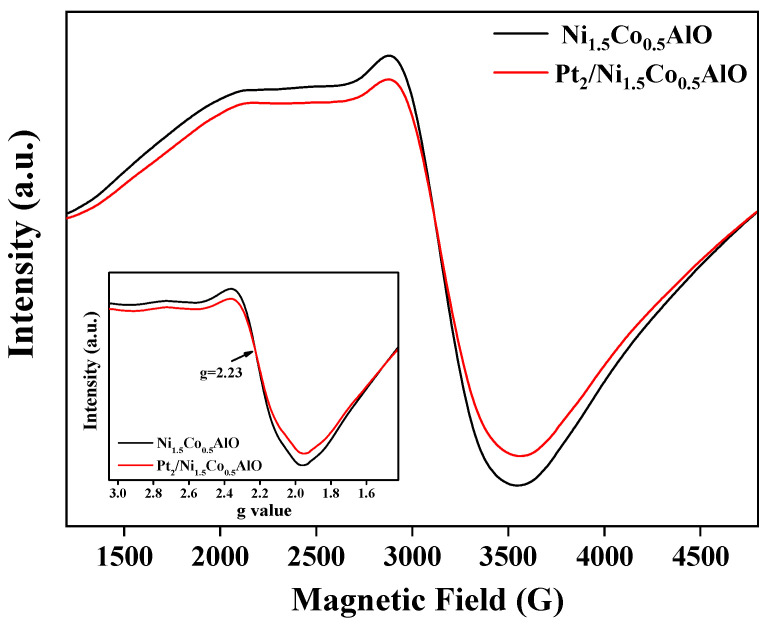
Electron paramagnetic resonance (EPR) spectra of Pt_n_/Ni_1.5_Co_0.5_AlO catalysts. The inset image exhibits the g value of the Pt_n_/Ni_1.5_Co_0.5_AlO catalysts.

The catalytic performance of NO oxidation is determined by the amounts of surface oxygen species. To investigate the essential relationship between Pt and dual Ni/Co cations and surface-reactive oxygen species, the electron paramagnetic resonance (EPR) spectrum was measured. EPR is a magnetic resonance technique that can study the microstructure and local environment around transition metal ions with an unpaired electron. The EPR spectrum can provide a detailed description of the electronic structure of the compound and the characteristics of the surrounding environment [55]. As shown in Figure 9, a single resolved band was observed in the Ni_1.5_Co_0.5_AlO catalyst, and the g value was located at ~2.23, which was assigned to the resonance absorption of paramagnetic Ni^2+^ ions [51,56]. After loading the Pt nanoparticles, the g value of the Pt_2_/Ni_1.5_Co_0.5_AlO catalyst was maintained at ~2.23, indicating that the resonance absorption peak was also mainly from Ni^2+^ ions. It is noted that the intensity of the EPR spectrum signal for the Pt_2_/Ni_1.5_Co_0.5_AlO catalyst was lower than that of the Ni_1.5_Co_0.5_AlO catalyst, indicating that supported Pt nanoparticles can affect the surrounding electron state of Ni^2+^ ions at the interface of Pt–support. As indicated by the results of XRD, FTIR, and XPS, the Co ions replace the positions of the Ni ions in the Pt_n_/Ni_1.5_Co_0.5_AlO catalysts, and the Pt atoms at the metal-oxide interface mainly exist as positively charged Pt^&+^ species. This result demonstrates that Pt atoms can mainly interact with Ni/Co atoms to promote the mobility of lattice oxygen and the formation of surface-oxygen vacancies. The synergistic effect of Pt and dual Ni/Co cations in the Pt/Ni_1.5_Co_0.5_AlO catalysts enhances the absorption and activation for gas reactants (NO and O_2_).

**Figure 10:**
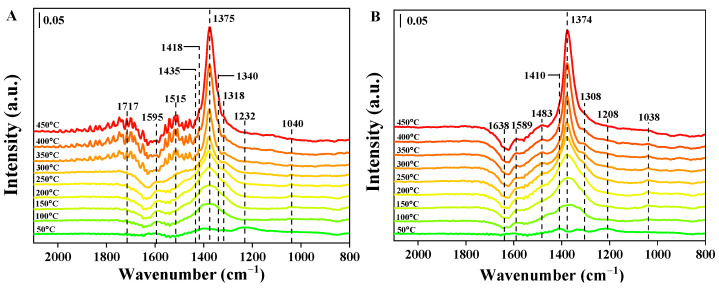
In situ DRIFT spectra of NO_x_ absorption on (**A**) the Ni_1.5_Co_0.5_AlO catalyst and (**B**) the Pt_2_/Ni_1.5_Co_0.5_AlO catalyst.

**Figure 11:**
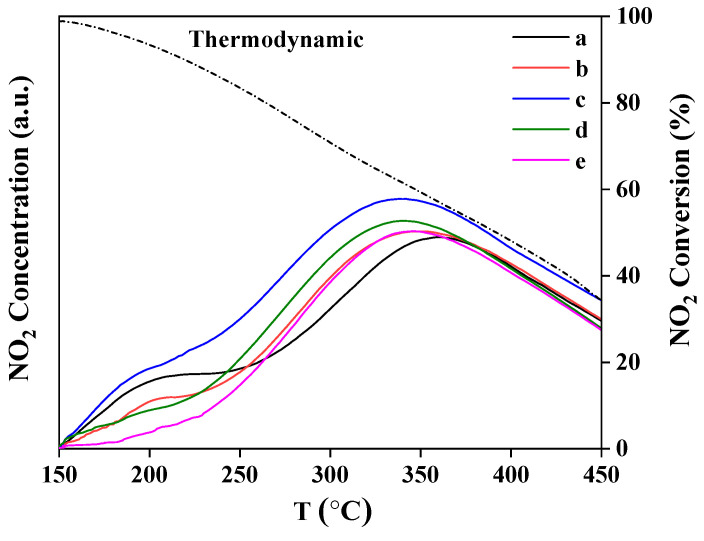
NO_2_ concentration curves of NO temperature-programmed oxidation over Pt_n_/Ni_1.5_Co_0.5_AlO catalysts: (a) Ni_1.5_Co_0.5_AlO; (b) Pt_1_/Ni_1.5_Co_0.5_AlO; (c) Pt_2_/Ni_1.5_Co_0.5_AlO; (d) Pt_4_/Ni_1.5_Co_0.5_AlO; (e) Pt_6_/Ni_1.5_Co_0.5_AlO.

The in situ diffuse reflectance infrared Fourier transform spectra (DRIFT) on the Pt_n_/Ni_1.5_Co_0.5_AlO catalysts were recorded to study the type of stored NO_x_ species. Figure 10 shows the in-situ resolved DRIFT spectra of the catalysts under NO and O_2_ atmosphere in the range from 50 to 450 °C, respectively. In the case of the Ni_1.5_Co_0.5_AlO catalysts (Figure 10A), the peaks were assigned as bridging nitrates (1595 cm^−1^), monodentate nitrates (1515 and 1318 cm^−1^), and bidentate nitrates (1232 and 1040 cm^−1^), respectively [57,58,59]. It is noted that the adsorption on the surface of Ni_1.5_Co_0.5_AlO support is mainly through-bridging nitrates (1595 cm^−1^) and bidentate nitrates (1232 cm^−1^) at 50 °C. As the temperature increased, the peaks emerged at 1595 cm^−1,^ assigned to bridging nitrates, and 1375 cm^−1^, assigned to bidentate nitrite (M-NO_2_), gradually increased. When the temperature increased to 350 °C, the peaks of the bridging nitrates (1595 cm^−1^) and the bidentate nitrates (1232 cm^−1^) gradually transformed to monodentate nitrates (1515 and 1318 cm^−1^). Several weakened peaks emerged at 1435, 1418, and 1340 cm^−1^, which were designated as nitrites. In addition, the peak at 1717 cm^−1^ was attributed to the adsorption of HNO_2_ [60]. These results demonstrated that the main adsorbed NO_x_ species on Ni_1.5_Co_0.5_AlO catalyst were monodentate nitrates and nitrate species at high temperatures (>300 °C). As shown in Figure 10B, after the loading of Pt nanoparticles, the peaks were designated as bridging nitrates (1589 cm^−1^), bidentate nitrate (1308 and 1038 cm^−1^), and monodentate nitrates (1483 cm^−1^) [53,54]. For NO and O_2_ adsorption at 50 °C, the peaks located at 1410 and 1208 cm^−1^ were ascribed to nitrites and bridging bidentate nitrites, respectively [61]. With the rising in temperature, the peaks at 1410 and 1208 cm^−1^ disappeared, while the intensity of those at 1589 and 1038 cm^−1^ increased gradually. The peak at 1038 cm^−1^ gradually vanished with the increase in temperature, while the intensity of the peak at 1308 cm^−1^ gradually increased and reached maximum intensity at 350 °C. In addition, the intensity of the peak at 1483 cm^−1^ increased with the rising in temperature, indicating that bidentate nitrates gradually transformed to bridging nitrates at high temperatures (>300 °C). At high temperatures (>300 °C), gaseous NO_2_ is generally considered to be produced by the decomposition of surface nitrite/nitrate species and free ion nitrate species [59]. Compared with the stretching frequency of NO_x_ species adsorbed at the surface of pure Ni_1.5_Co_0.5_AlO support, as shown in Figure 11A,B, there is a certain deviation of the Pt-supported catalyst. It has been reported that the strength of NO adsorption and NO stretching frequency are closely related to the electronic structure of metal [62]. It is suggested that these deviations are attributed to the strong interaction between Pt nanoparticles and support. These results are consistent with the NO-TPO measurement and further verify the NO_2_-assisted mechanism to boost soot combustion.

## 4. Discussion

The catalytic combustion of soot particles is a typical three-phase complex deep-oxidation reaction of solid (catalysts)–solid (soot particles)–gas (reactant gas). The solid–solid contact condition is an important rate-determining factor. Pt nanoparticles are uniformly dispersed on Ni_1.5_Co_0.5_AlO nanosheets by the GBMR method, and the regular morphology of the nanosheets is maintained. The specific structure formed by the stacking of hydrotalcite-like Ni_1.5_Co_0.5_AlO nanosheets synthesized by the urea hydrothermal method can improve the mass transfer efficiency between the catalysts and the gaseous reactants (NO and O_2_). The strong interaction between Pt and Ni_1.5_Co_0.5_AlO can weaken the Ni/Co-O bond to increase the mobility of lattice oxygen and facilitate the formation of oxygen vacancies at the interface of Pt–support. The synergistic effect of Pt and dual Ni/Co cations in the Pt/Ni_1.5_Co_0.5_AlO catalysts improves the adsorption and activation characteristics of the gas reactants to boost soot combustion. 

The evaluation of NO oxidation abilities over the Pt_n_/Ni_1.5_Co_0.5_AlO catalysts was measured by NO-TPO profiles. As shown in Figure 11, the NO_2_ concentration peak of Ni_1.5_Co_0.5_AlO support emerged at 365 °C, while the Pt-based catalysts shifted toward low temperature. The NO_2_ concentration did not increase with increasing temperature until the thermodynamic equilibrium of the equation (NO+1/2 O_2_ ⇄ NO_2_) was satisfied, and then it decreased at higher temperatures [34]. In addition, the NO_2_ concentration peak of the Pt_1_/Ni_1.5_Co_0.5_AlO catalyst shifted to 350 °C. With the increase in Pt content, the peak of Pt_2_/Ni_1.5_Co_0.5_AlO catalyst located at 338 °C, indicating that the synergistic effect of Pt and dual Ni/Co cations in the Pt_n_/Ni_1.5_Co_0.5_AlO catalysts can enhance the ability of adsorption and activation for O_2_ and promote NO oxidation. With further increases in Pt content, the NO_2_ concentration peaks of the Pt_4_/Ni_1.5_Co_0.5_AlO and Pt_6_/Ni_1.5_Co_0.5_AlO catalysts were located at 340 and 343 °C, respectively. In the process of catalytic oxidation, the concentration of NO_2_ is closely related to the oxidation rate of soot particles [63]. The intensity of NO_2_ concentration peak over the Pt_2_/Ni_1.5_Co_0.5_AlO catalyst possessed the highest value, indicating that the Pt_2_/Ni_1.5_Co_0.5_AlO catalyst had the best catalytic performance during soot combustion. This result is consistent with soot-TPO measurement. 

**Figure 12:**
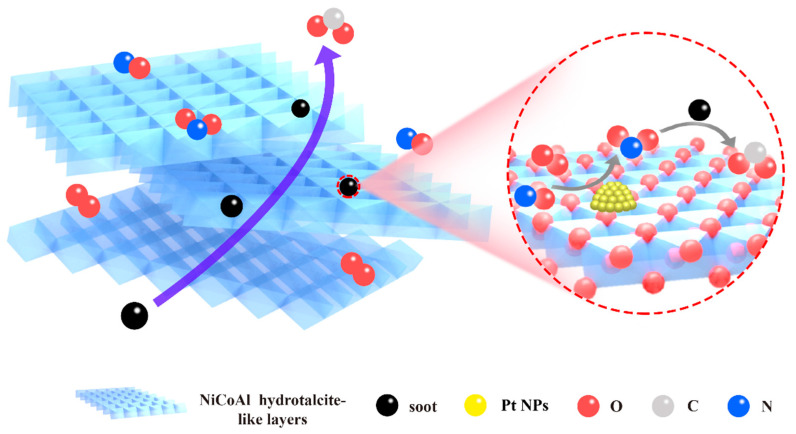
Mechanism diagram of the Pt_n_/Ni_1.5_Co_0.5_AlO catalysts for soot combustion under loose-contact conditions.

On the basis of the above results and discussions, Figure 12 vividly displays the pathway of soot oxidation over the Pt_n_/Ni_1.5_Co_0.5_AlO catalysts. First, the uniform dispersion of the Pt nanoparticles on the hydrotalcite-like nanosheets of Ni_1.5_Co_0.5_AlO support enhanced the contact efficiency between the soot and the catalysts. Second, the effective transfer mass efficiency between the reactants (O_2_, NO, and soot) was achieved by the specific pore structure formed by the stacking of the hydrotalcite-like nanosheets. Third, combining the results of H_2_-TPR, XPS, and EPR, the strong interaction of Pt-Ni_1.5_Co_0.5_AlO weakened the Ni/Co-O bond for promoting the mobility of lattice oxygen and the formation of surface-oxygen vacancies at the interface of Pt–support, which is beneficial for improving the adsorption and activation of O_2_ molecules, and thus increasing the amounts of reactive oxygen species. The surface-active oxygen species can significantly promote the oxidation of NO to NO_2_ intermediates. Finally, the NO_2_ transferred from the Pt-Ni_1.5_Co_0.5_AlO interface to the surface of the soot particles and oxidized them to CO and CO_2_. (overall reaction: NO + 1/2O_2_ → NO_2_ and NO_2_ + soot → CO + CO_2_). Pt and dual Ni/Co cations in the Pt/Ni_1.5_Co_0.5_AlO catalysts achieved synergistic catalytic soot combustion. Therefore, the Pt_n_/Ni_1.5_Co_0.5_AlO catalysts exhibited excellent catalytic performance to boost soot combustion.

## 5. Conclusions

In this paper, Pt_n_/Ni_1.5_Co_0.5_AlO catalysts were elaborately fabricated by the GBMR method. The specific porous structure formed by the stack of hydrotalcite-derived Ni_1.5_Co_0.5_AlO nanosheets can increase the transfer mass efficiency of the reactants (O_2_, NO, and soot), and the strong Pt-Ni_1.5_Co_0.5_AlO interaction can weaken the Ni/Co-O bond for promoting the mobility of lattice oxygen and the formation of surface oxygen vacancies. Pt_n_/Ni_1.5_Co_0.5_AlO catalysts exhibited excellent catalytic activity and stability during soot combustion. Among all the catalysts, the Pt_2_/Ni_1.5_Co_0.5_AlO catalyst showed the highest catalytic activities for soot combustion, i.e., the values of *T_50_*, TOF, and *Ea* were 350 °C, 6.63 × 10^−3^ s^−1^, and 57.1 kJ mol^−1^, respectively. Based on the characterization results, the catalytic mechanism for soot combustion was proposed: the synergistic effect of Pt and dual Ni/Co cations in Pt/Ni_1.5_Co_0.5_AlO catalysts can promote the vital step of catalyzing NO oxidation to NO_2_ in the NO-assisted soot oxidation mechanism. The in-depth understanding of the catalytic mechanism for Pt_n_/Ni_1.5_Co_0.5_AlO catalysts is meaningful for the development of high-efficient catalysts in practical applications during soot combustion.

## Figures and Tables

**Table 1:** Catalytic activities, densities of active oxygen, relative reaction rates (R), and TOF values of Pt_n_/Ni_1.5_Co_0.5_AlO catalysts for soot combustion under conditions of loose contact.

Catalysts	*T_10_* (°C)	*T_50_* (°C)	*T_90_* (°C)	S_CO2_^m^ (%)	Oxygen Amount (µmol g^−1^)	Density of Oxygen (µmol g^−1^)	R (µmol g^−1^ s^−1^)	TOF (10^−3^ s^−1^)
soot	461	584	648	65.2	-	-	-	-
Ni_1.5_Co_0.5_AlO	320	380	419	99.4	12.78	25.56	0.12	4.69
Pt_1_/ Ni_1.5_Co_0.5_AlO	287	368	404	99.7	13.18	26.37	0.14	5.30
Pt_2_/ Ni_1.5_Co_0.5_AlO	260	350	383	99.3	15.84	31.68	0.21	6.63
Pt_4_/ Ni_1.5_Co_0.5_AlO	267	356	393	99.1	14.67	29.35	0.17	5.79
Pt_6_/ Ni_1.5_Co_0.5_AlO	265	358	398	99.1	13.54	27.08	0.16	5.54

## Data Availability

Not applicable.

## Supplementary Materials

The following supporting information can be downloaded at: https://www.mdpi.com/article/10.3390/nano13040623/s1: Experimental Section: 1.1 Detailed information of catalytic activity evaluation; Figure S1: Schematic representation of gas bubbling-assisted membrane reduction (GBMR) device; Figure S2: Raman spectra of Ni1.5Co0.5AlO and Pt6/Ni1.5Co0.5AlO catalysts; Figure S3: XPS spectra of Al 2p regions over Pt_n_/Ni_1.5_Co_0.5_AlO catalysts. (a) Ni_1.5_Co_0.5_AlO; (b) Pt_1_/Ni_1.5_Co_0.5_AlO; (c) Pt_2_/Ni_1.5_Co_0.5_AlO; (d) Pt_4_/Ni_1.5_Co_0.5_AlO; (e) Pt_6_/Ni_1.5_Co_0.5_AlO; Figure S4: Catalytic performances for soot combustion over Pt_n_/Ni_1.5_Co_0.5_AlO catalysts. (a) Ni_1.5_Co_0.5_AlO; (b) Pt_1_/Ni_1.5_Co_0.5_AlO; (c) Pt_2_/Ni_1.5_Co_0.5_AlO; (d) Pt_4_/Ni_1.5_Co_0.5_AlO; (e) Pt_6_/Ni_1.5_Co_0.5_AlO; (f) no catalyst; Figure S5: The variation curves of CO_2_ concentration changed with time during the soot combustion over Pt_n_/Ni_1.5_Co_0.5_AlO catalysts; Figure S6: The stability test for soot combustion (A) and soot conversion (B) over Pt_2_/Ni_1.5_Co_0.5_AlO catalyst under the loose contact condition; Figure S7: Catalytic performances for soot combustion (A) and soot conversion (b) over of Ni_1.5_Co_0.5_AlO catalyst in O_2_ (5%) balanced with Ar gas under the loose contact condition; Table S1: BET surface areas (S_BET_), pore volume (V_p_), pore diameter (D_p_) of Pt_n_/Ni_1.5_Co_0.5_AlO catalysts; Table S2: Surface compositions and oxidation states of Pt, Co, Ni, O and Al species over Ptn/Ni1.5Co0.5AlO catalysts derived from XPS analyses; Table S3: The apparent activation energy *E_a_* for NO_x_-assisted soot combustion over Pt_n_/Ni_1.5_Co_0.5_AlO catalysts.
