# Supplementary material for: Synergistic Effect of Pt and Dual Ni/Co Cations in Hydrotalcite-Derived Pt/Ni1.5Co0.5AlO Catalysts for Promoting Soot Combustion
Source: Nanomaterials (Basel). 2023 Feb 4;13(4):623. doi: 10.3390/nano13040623 (PMC9965507; doi:10.3390/nano13040623)
Supplement: Supplementary file 1 [file nanomaterials-13-00623-s001.zip › nanomaterials-2158379-supplementary.pdf]

Supporting Information

Article

Synergistic Effect of Pt and Dual Ni/Co Cations in Hydrotalcite-Derived Pt/$Ni_{1.5}Co_{0.5}AlO$ Catalysts for Promoting Soot Combustion

Yilin Zhang [1], Peng Zhang [1], Jing Xiong [1,2], Yuanfeng Li [1], Yaxiao Ma [1], Sicheng Zhang [1], Zhen Zhao [1], Jian Liu [1]
and Yuechang Wei [1,2,*]

1 State Key Laboratory of Heavy Oil Processing, College of Science, China University of Petroleum, Beijing, 102249, China; zhangyilin312@126.com (Y.Z.); zhangpeng_cup@aliyun.com (P.Z.); cup_xiongjing@163.com (J.X.); liyuanfeng@sust.edu.cn (Y.L.); mayaxiao2021@163.com (Y.M.); zhangsc0211@163.com (S.Z.); zhenzhao@cup.edu.cn (Z.Z.); liujian@cup.edu.cn (J.L.)
2 Key Laboratory of Optical Detection Technology for Oil and Gas, China University of Petroleum, Beijing, 102249, China
* Correspondence: weiyc@cup.edu.cn

Figures and Table Captions

1. Experimental Section

1.1 Detailed information of catalytic activity evaluation

Fig. S1. Schematic representation of gas bubbling-assisted membrane reduction (GBMR) device.

Fig. S2. Raman spectra of $Ni_{1.5}Co_{0.5}AlO$ and $Pt_6/Ni_{1.5}Co_{0.5}AlO$ catalysts.

Fig. S3. XPS spectra of Al 2p regions over $Pt_n/Ni_{1.5}Co_{0.5}AlO$ catalysts. (a) $Ni_{1.5}Co_{0.5}AlO$; (b) $Pt_1/Ni_{1.5}Co_{0.5}AlO$; (c) $Pt_2/Ni_{1.5}Co_{0.5}AlO$; (d) $Pt_4/Ni_{1.5}Co_{0.5}AlO$; (e) $Pt_6/Ni_{1.5}Co_{0.5}AlO$

Fig. S4. Catalytic performances for soot combustion over $Pt_n/Ni_{1.5}Co_{0.5}AlO$ catalysts.

(a) $Ni_{1.5}Co_{0.5}AlO$; (b) $Pt_1/Ni_{1.5}Co_{0.5}AlO$; (c) $Pt_2/Ni_{1.5}Co_{0.5}AlO$; (d) $Pt_4/Ni_{1.5}Co_{0.5}AlO$; (e) $Pt_6/Ni_{1.5}Co_{0.5}AlO$; (f) no catalyst

Fig. S5. The variation curves of CO_2 concentration changed with time during the soot combustion over $Pt_n/Ni_{1.5}Co_{0.5}AlO$ catalysts.

Fig. S6. The stability test for soot combustion (A) and soot conversion (B) over $Pt_2/Ni_{1.5}Co_{0.5}AlO$ catalyst under the loose contact condition.

Fig. S7. Catalytic performances for soot combustion (A) and soot conversion (b) over of $Ni_{1.5}Co_{0.5}AlO$ catalyst in O_2 (5%) balanced with Ar gas under the loose contact condition.

Table S1. BET surface areas (S_{BET}), pore volume (V_p), pore diameter (D_p) of $Pt_n/Ni_{1.5}Co_{0.5}AlO$ catalysts.

Table S2. Surface compositions and oxidation states of Pt, Co, Ni, O and Al species over $Pt_n/Ni_{1.5}Co_{0.5}AlO$ catalysts derived from XPS analyses.

Table S3. The apparent activation energy E_a for NO_x-assisted soot combustion over $Pt_n/Ni_{1.5}Co_{0.5}AlO$ catalysts.

1. Experimental Section

1.1 Detailed information of catalytic activity evaluation

Turnover frequency (TOF) is defined as the ratio of reaction rate (*R*) to surface active oxygen density. When the conversion rate of soot is in a stable and appropriate conversion rate (less than 10 %), it can be approximately considered within the approximate kinetic range. Due to the low conversion of soot oxidation and almost constant change over time, the isothermal reaction and isothermal anaerobic titration temperature is selected in the kinetic range of about 300 °C, the reaction is carried out under the conditions of 5% O_2 and 0.2% NO, using Ar as the equilibrium gas, and the total flow rate is 50 mL min^{-1}. The relative conversion rate (*R*) of $Pt_n/Ni_{1.5}Co_{0.5}AlO$ catalysts can be obtained by following detailed method: The T_{10} value of catalyst can be obtained by soot-TPO, and the conversion rate (*R*) of catalyst is obtained through isothermal reaction for soot oxidation. We keep the temperature of the quartz tube at T_{10} and continuously obtain the outlet CO and CO_2 concentration. The amount of soot conversion can be obtained by the following equation:

$$C_{soot}=[C_{CO}+C_{CO2}]\cdot Q/[22400\cdot m]$$

Here, the $[C_{CO}]$ and $[C_{CO2}]$ represents the CO and CO_2 concentration, respectively, measured by isothermal reactions, the Q represents the gas flow rate 50 mL min^{-1} and m represents the quality of catalyst (0.1g). Finally, we can obtain the relationship between soot conversion amount and time, and can perform linear fitting to obtain the slope of the line. And the slope of the line is the conversion rates for $Pt_n/Ni_{1.5}Co_{0.5}AlO$ catalysts.

The amount and density of reactive oxygen species on the catalyst surface is obtained by isothermal anaerobic titration using soot particles as probe molecules. are calculated by isothermal anaerobic titration. Instantaneous removal of O_2 feed, monitoring the transient attenuation of steady-state CO_2 concentration. Since no further oxygen was added into the reaction system, the amount of stored oxygen can be calculated from the subsequent total CO_2

production. O* was calculated according to one CO_2 molecule generated by two O* atoms. The amount of reactive oxygen species can be obtained by the following equation:

$$\text{O* amount } (\mu\text{mol g}^{-1}) = 2*10^{-6}*P_0*V*A/(R*T*m)$$

Here, P_0 is the atmospheric pressure, Pa; V is the volumetric flow rate, m^3 s^{-1}; A is the integral of CO_2 concentration curves as a function of time during the isothermal anaerobic titration, s; R represents the gas constant; T represents the reaction temperature, K; and m represents the catalyst weight, g. The density of active oxygen (D_o) is equal to 2 times the consumed active oxygen amount.

The apparent activation energy (Ea) was calculated from the least squares fit of $\ln\left[-\frac{\ln(1-\alpha)}{T^2}\right]$ and versus $\frac{1}{T}$ data series, using the following equation[1]:

$$\ln\left[-\frac{\ln(1-\alpha)}{T^2}\right] = \ln\left[\frac{AR}{\beta E}\left(1-\frac{2RT}{E}\right)\right] - \frac{E}{R}\cdot\frac{1}{T}$$

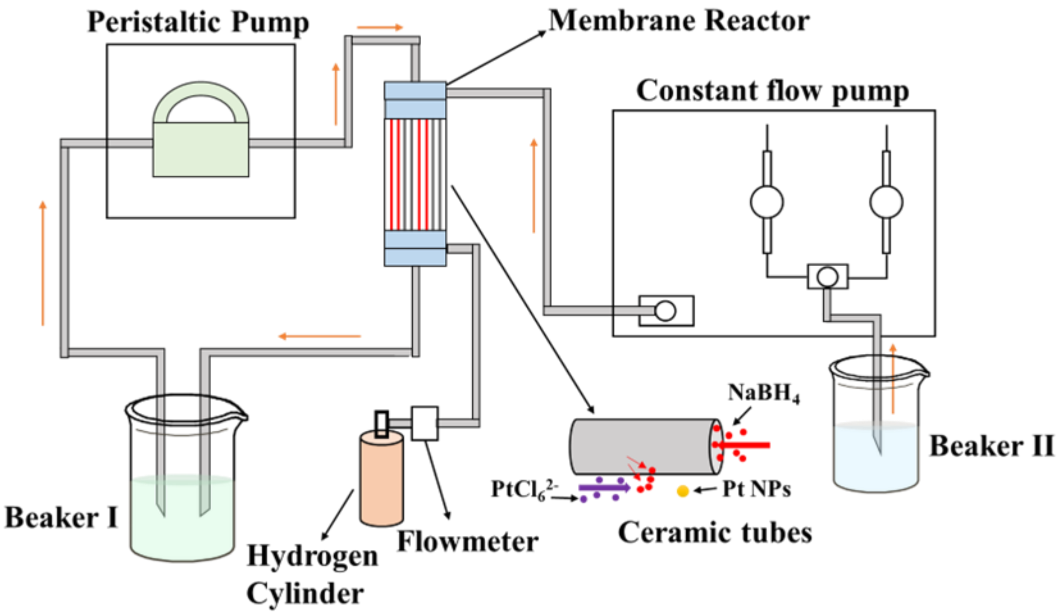

Fig. S1. Schematic representation of gas bubbling-assisted membrane reduction (GBMR) device.

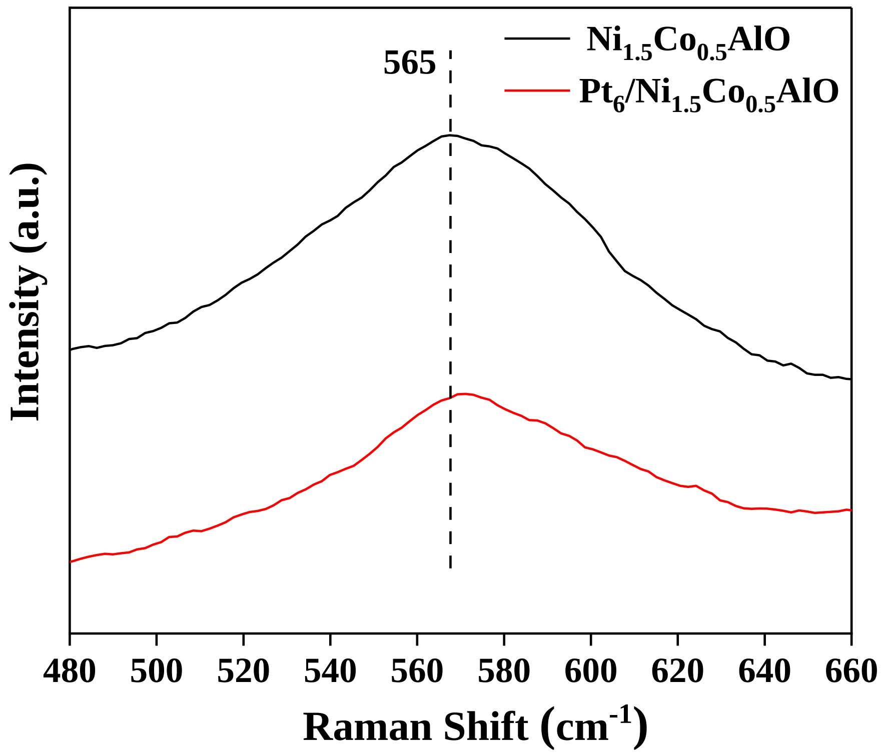

Fig. S2. Raman spectra of $Ni_{1.5}Co_{0.5}AlO$ and $Pt_6/Ni_{1.5}Co_{0.5}AlO$ catalysts.

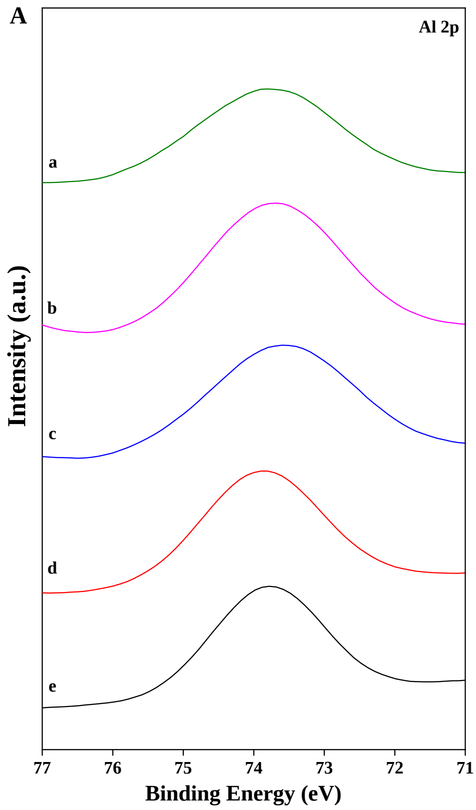

Fig. S3. XPS spectra of Al 2p regions over $Pt_n/Ni_{1.5}Co_{0.5}AlO$ catalysts. (a) $Ni_{1.5}Co_{0.5}AlO$; (b) $Pt_1/Ni_{1.5}Co_{0.5}AlO$; (c) $Pt_2/Ni_{1.5}Co_{0.5}AlO$; (d) $Pt_4/Ni_{1.5}Co_{0.5}AlO$; (e) $Pt_6/Ni_{1.5}Co_{0.5}AlO$

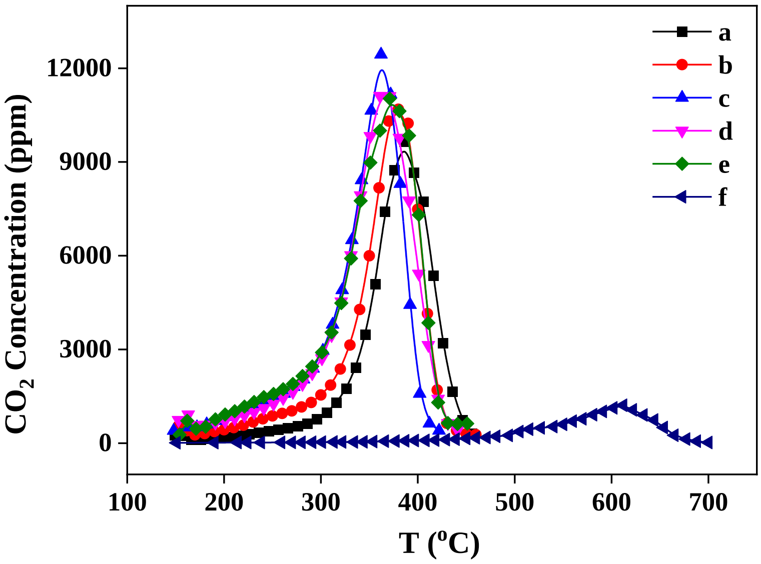

Fig. S4. Catalytic performances for soot combustion over $Pt_n/Ni_{1.5}Co_{0.5}AlO$ catalysts. (a) $Ni_{1.5}Co_{0.5}AlO$; (b) $Pt_1/Ni_{1.5}Co_{0.5}AlO$; (c) $Pt_2/Ni_{1.5}Co_{0.5}AlO$; (d) $Pt_4/Ni_{1.5}Co_{0.5}AlO$; (e) $Pt_6/Ni_{1.5}Co_{0.5}AlO$; (f) no catalyst

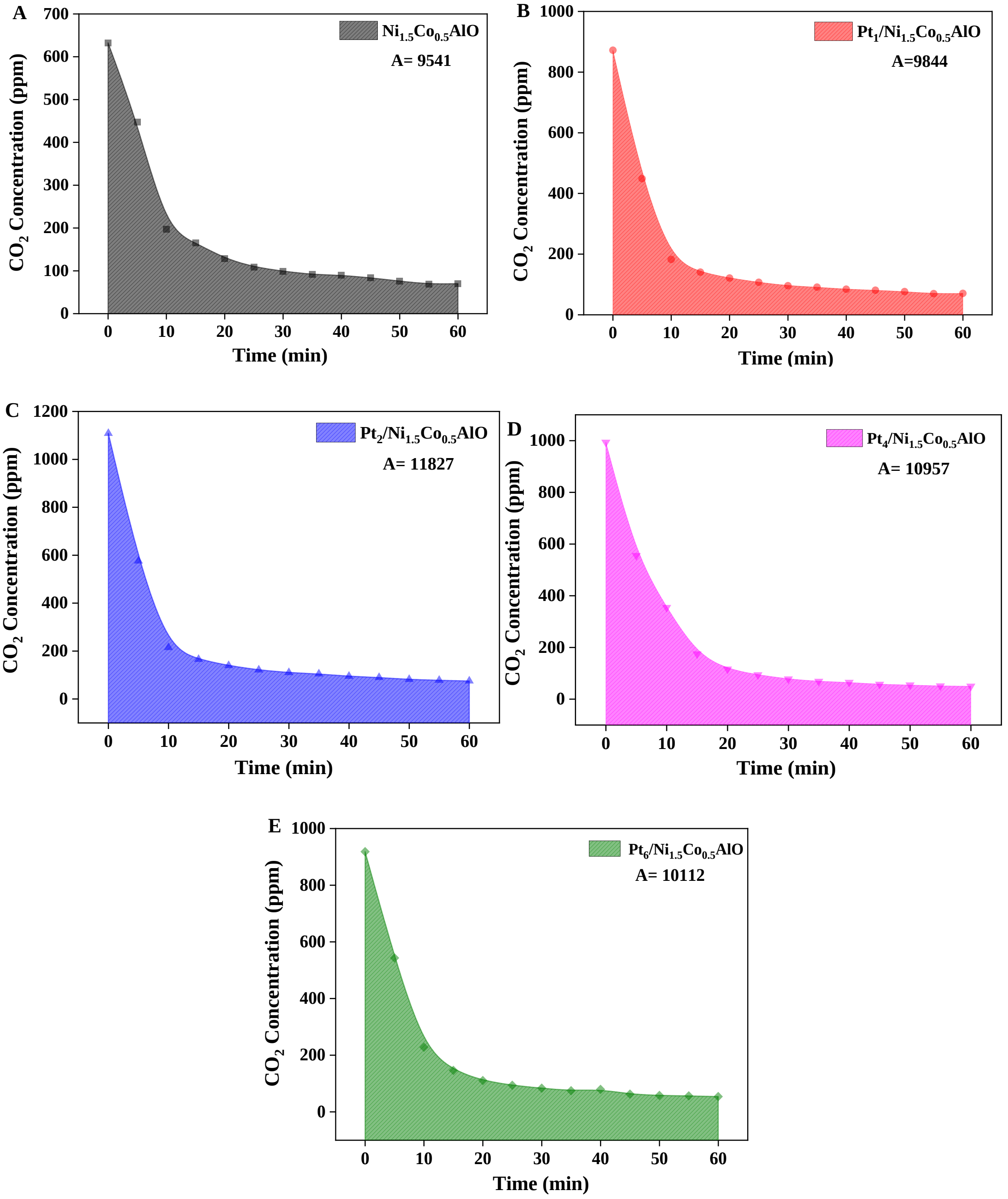

Fig. S5. The variation curves of CO_2 concentration changed with time during the soot combustion over $Pt_n/Ni_{1.5}Co_{0.5}AlO$ catalysts (A)***;(B)***;(C)***;(D)***;(E)***.

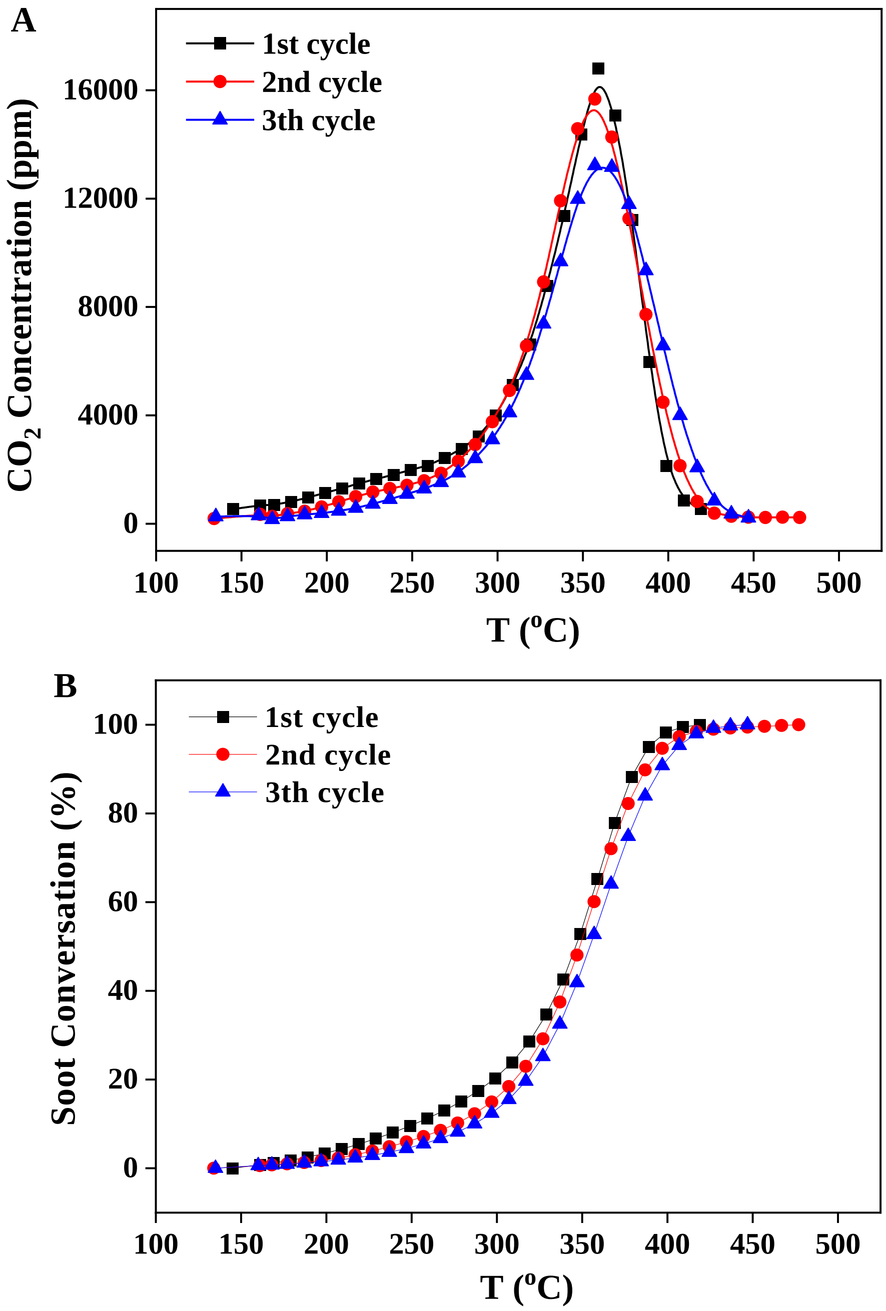

Fig. S6. The stability test for soot combustion (A) and soot conversion (B) over $Pt_2/Ni_{1.5}Co_{0.5}AlO$ catalyst under the loose contact condition.

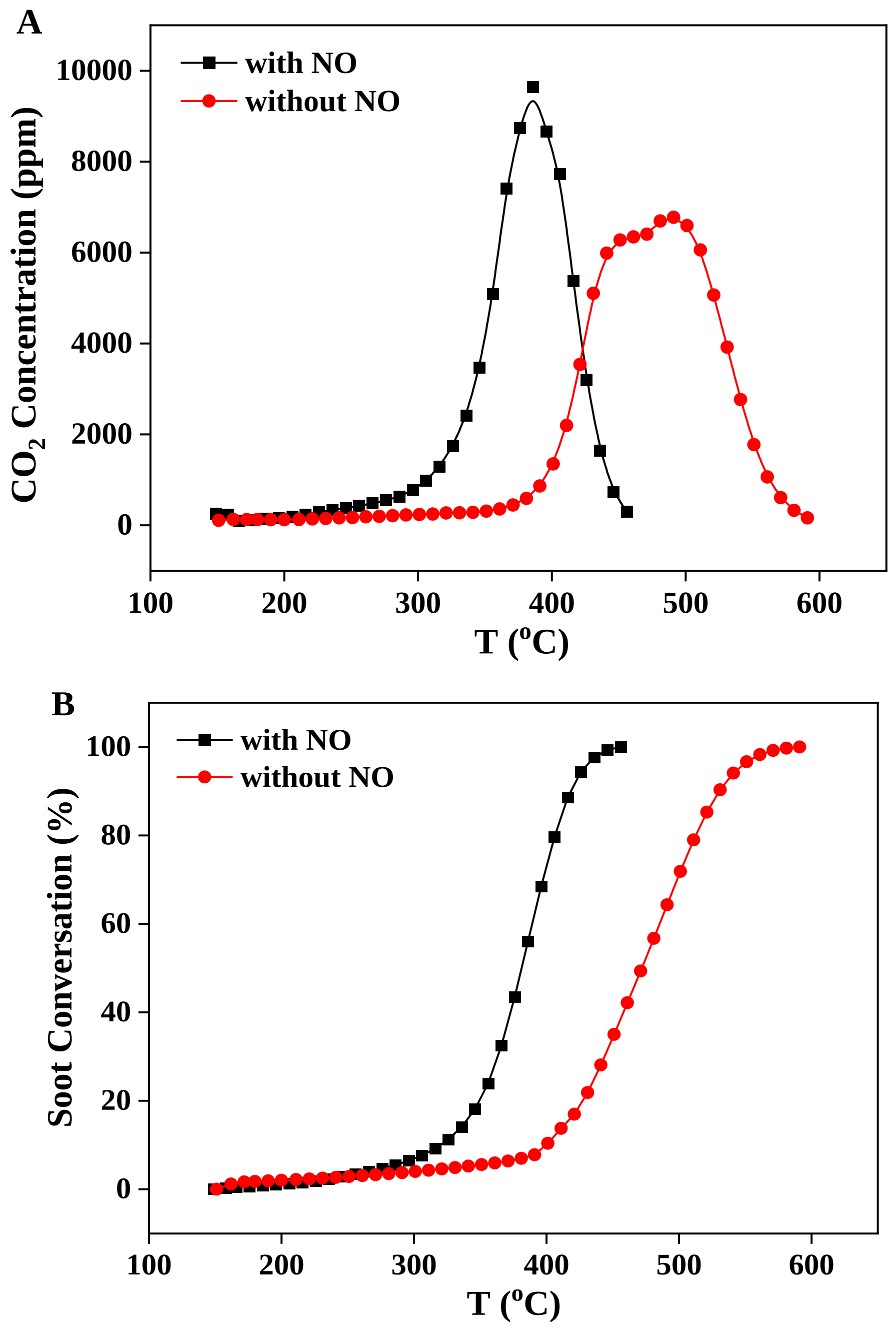

Fig. S7. Catalytic performances for soot combustion (A) and soot conversion (b) over of $Ni_{1.5}Co_{0.5}AlO$ catalyst in O_2 (5%) balanced with Ar gas under the loose contact condition.

Table S1. BET surface areas (S_{BET}), pore volume (V_P), pore diameter (D_P) of $Pt_n/Ni_{1.5}Co_{0.5}AlO$ catalysts.

**Catalysts**	**Surface Area (m^2 g^{-1}) [a]**	**Total Pore Volume (cm^3 g^{-1}) [b]**	**Average Pore Diameter (nm) [c]**
$Ni_{1.5}Co_{0.5}AlO$	189.06	0.40	7.12
$Pt_1/Ni_{1.5}Co_{0.5}AlO$	123.72	0.31	7.95
$Pt_2/Ni_{1.5}Co_{0.5}AlO$	122.18	0.34	7.84
$Pt_4/Ni_{1.5}Co_{0.5}AlO$	124.84	0.29	7.59
$Pt_6/Ni_{1.5}Co_{0.5}AlO$	123.31	0.28	6.37

[a] Surface area were calculated by the BET method.

[b] Pore volume were obtained by the BET method.

[c] Pore diameter was calculated through the BJH method based on the N_2 desorption isotherms.

Table S2. Surface compositions and oxidation states of Pt, Co, Ni, O and Al species over $Pt_n/Ni_{1.5}Co_{0.5}AlO$ catalysts derived from XPS analyses.

Catalysts	Pt species (%)				Co species (%)			Ni species (%)			O species (%)			
	Pt^0	Pt^{2+}	Pt^{4+}	*R* [a]	Co^{3+}	Co^{2+}	*R* [b]	Ni^{3+}	Ni^{2+}	*R* [c]	O^{2-}	O_2^{2-} /O_2^-	CO_3^{2-} /-OH	*R* [d]
$Ni_{1.5}Co_{0.5}AlO$	-	-	-	-	63.4	36.6	1.732	67.3	32.7	2.058	49.9	37.7	12.4	1.004
$Pt_1/Ni_{1.5}Co_{0.5}AlO$	31.8	14.9	53.3	2.145	63.2	36.8	1.717	65.7	34.3	1.915	48.9	32.0	19.1	1.044
$Pt_2/Ni_{1.5}Co_{0.5}AlO$	31.1	16.2	52.7	2.215	62.3	37.7	1.653	64.3	35.7	1.801	46.9	33.7	19.4	1.132
$Pt_4/Ni_{1.5}Co_{0.5}AlO$	31.3	16.8	51.9	2.195	62.5	37.5	1.667	67.1	32.9	2.039	47.5	30.9	21.6	1.105
$Pt_6/Ni_{1.5}Co_{0.5}AlO$	31.4	14.2	54.4	2.185	62.6	37.4	1.673	67.2	32.8	2.048	48.0	35.9	16.1	1.083

[a] The Pt species ratio of $Pt^{\&+}$(Pt^{2+} + Pt^{4+}) to Pt^0.

[b] The Co species ratio Co^{3+}/Co^{2+}.

[c] The Ni species ratio Ni^{3+}/Ni^{2+}.

[d] The ratio of the absorbed oxygen (O_2^{2-}/O_2 + CO_3^{2-}/-OH) to lattice oxygen (O^{2-}).

Table S3. The apparent activation energy E_a for NO_x-assisted soot combustion over $Pt_n/Ni_{1.5}Co_{0.5}AlO$ catalysts.

**Catalysts**	**Slope**	**Correlation Coefficients (R^2)**	**Apparent Activation Energy (E_a) (kJ mol^{-1})**
$Ni_{1.5}Co_{0.5}AlO$	-8.01	0.999	66.6
$Pt_1/Ni_{1.5}Co_{0.5}AlO$	-7.70	0.999	64.1
$Pt_2/Ni_{1.5}Co_{0.5}AlO$	-6.87	0.986	57.1
$Pt_4/Ni_{1.5}Co_{0.5}AlO$	-7.18	0.997	59.7
$Pt_6/Ni_{1.5}Co_{0.5}AlO$	-7.33	0.994	60.9

References

1. Zhao, M.; Deng, J.; Liu, J.; Li, Y.; Liu, J.; Duan, Z.; Xiong, J.; Zhao, Z.; Wei, Y.; Song, W.; Sun, Y. Roles of Surface-Active Oxygen Species on 3DOM Cobalt-Based Spinel Catalysts $M_xCo_{3-x}O_4$ (M = Zn and Ni) for NO_x-Assisted Soot Oxidation. *ACS Catal.* 2019, 9, 7548-7567.
